# Quantum-enhanced deep learning for elastographic image-based characterization of cutaneous and subcutaneous masses

**DOI:** 10.3389/fmed.2026.1925092

**Published:** 2026-09-11

**Authors:** Hongping Zhang

**Affiliations:** The People's Hospital of Jianyang City, Jianyang, Chengdu, Sichuan, China

**Keywords:** cutaneous masses, elastography, quantum machine learning, soft-tissue tumors, variational quantum circuits

## Abstract

**Objective:**

Improving the diagnostic precision of skin and subcutaneous lesion assessments is the main goal of this study. Although elastography is an effective method for determining tissue stiffness, the high-dimensional, non-linear data these scans generate frequently present challenges for traditional deep learning models. To capture fine-grained stiffness patterns that conventional models may overlook, this study proposes and evaluates a hybrid quantum-classical deep learning architecture.

**Methods:**

A retrospective dataset of 520 elastographic images with histological confirmation is used in the investigation. The proposed methodology uses a multi-stage hybrid pipeline, in which initial feature vectors are extracted from raw images using a traditional Convolutional Neural Network (CNN). A parameterized quantum circuit layer receives these vectors. This layer recognizes complex textural patterns by performing extensive transformations within a high-dimensional Hilbert space. A final fully connected layer processes the output to categorize lesions as either benign or malignant. We present a proof-of-concept simulation of combining a parameterized quantum layer with a classical CNN backbone. Although the current classical software emulation introduces a latency trade-off (124.5 ms vs. 8.4 ms per image), our result can serve as a baseline for analyzing low-parameter feature representations before running on physical QPU hardware.

**Results:**

Compared to fully classical baselines, the hybrid model showed a notable performance improvement. Important conclusions include 94.3% diagnostic accuracy, 93.8% sensitivity, and 94.7% specificity. From 0.921 (classical) to 0.963 (hybrid), the Area Under the ROC Curve (AUC) rose by 4.2%. Testing revealed that, especially in situations with unclear stiffness patterns, the quantum layer was the main source of increased predictive power.

**Discussion:**

Quantum circuit integration offers more “expressive power” than conventional neural networks. The model efficiently captures non-linear correlations in tissue density that are typically flattened or ignored by classical architectures, as it operates in a high-dimensional Hilbert space. This implies that combining quantum and classical techniques is especially well-suited to medical imaging, where minute changes in data have a clinically significant impact.

**Conclusion:**

The study concludes that when it comes to describing cutaneous and subcutaneous masses, the hybrid classical-quantum model performs noticeably better than conventional deep learning techniques. These results indicate a promising advancement in medical AI that could provide physicians with more accurate and reliable tools for the early identification of cancer using elastographic data.

## 1 Introduction

Lesions of the skin and subcutaneous tissues that present as soft-tissue masses are a diverse group of abnormalities and are commonly seen in general surgery, dermatology, and oncology. The incidence of soft-tissue sarcomas worldwide is 5 per 100,000 people per year; however, benign lesions such as lipomas and epidermoid cysts predominate, with a population prevalence greater than 2% (1, 2). Non-invasive differentiation between malignant and benign masses is also of great clinical relevance: over-excision of benign masses may lead to increased patient morbidity, while delayed diagnosis of malignant tumors is associated with worse prognosis (3). A broad spectrum of lesions varies from common benign cysts and lipomas to malignant neoplasms such as melanoma, squamous cell carcinoma, and metastases (4). The above lesions need to be accurately characterized for appropriate clinical management; however, their characterization is confounded by both subjectivity in clinical palpation and by common morphological features (5). Conventional diagnostic methods rely on visual examination, palpation, and, ultimately, histopathology of biopsy specimens, which are invasive, time-consuming, and associated with procedural risks (6). Elastography is a novel noninvasive imaging technique that maps tissue stiffness, providing additional information beyond traditional ultrasonography (7). The basis of elastography is that malignant tissues are stiffer than benign lesions due to increased cellular density, a desmoplastic reaction, and a modified extracellular matrix composition (8).

Different elastographic techniques have been described, such as elastography, which estimates tissue strain under mechanical compression, and shear-wave elastography, which measures the velocity of shear waves induced by acoustic radiation force impulses (9, 10). Clinical studies have confirmed that elastography helps differentiate benign and malignant skin and subcutaneous masses, in which malignant lesions exhibit significantly higher stiffness values (11, 12). It is well established that Ultrasound (US) is the modality of choice for superficial masses because it is widely available, offers real-time evaluation, and does not use ionizing radiation. However, standard B-mode US provides poor contrast for differentiation of lesion subtypes with similar echogenicity. Ultrasound elastography overcomes this barrier by measuring tissue mechanical properties, with tissue malignancy strongly correlated with these parameters, particularly shear-wave velocity and strain ratio, as cancerous tissue tends to have 2-10 times higher stiffness than adjacent normal tissue. However, interpretation of elastographic images remains operator-dependent and varies among observers. Deep learning-based computer-aided diagnosis systems have demonstrated potential for analyzing medical images, such as elastography.

DL has achieved unprecedented success in medical image analysis, including lesion segmentation, classification, and prognostication. Convolutional neural networks (CNNs) applied to elastographic images can automatically derive stiffness-related texture features, achieving ideal performance for all radiologist observers on benchmark datasets. Nevertheless, traditional deep learning models are fundamentally limited in their ability to model the high-dimensional, nonlinear characteristics of elastographic data, and their performance tends to saturate as model complexity increases. Quantum computing (13) represents a paradigm shift in computation, with provable improvements in certain high-dimensional learning tasks. Quantum Machine Learning (QML) (14) utilizes superposition and entanglement to encode and operate on large feature spaces using polynomial qubit counts. Quantum machine learning (QML) has recently been proposed as a method that leverages quantum computing principles to process information in ways that classical computing cannot efficiently simulate (16). Variational Quantum Circuits (VQCs) (15), also known as quantum neural networks, are parameterized circuits that can be trained using hybrid classical-quantum optimization techniques and have been shown to perform competitively on image classification tasks (17). Parameterized quantum circuits (PQCs) can be combined with classical neural networks to form hybrid quantum-classical neural network architectures, with the potential to improve representational power and computational efficiency (18). Initial QML implementations in medical imaging are promising for applications such as lung cancer classification, breast cancer detection, and brain tumor segmentation (19–21).

Recent developments in deep learning have led to significant improvements in the representation of medical images in both ultrasound and surgical vision (22). Deep learning models for ultrasound localization microscopy (ULM) (23) have recently been introduced to enable rapid super-resolution microvessel imaging by directly localizing high-density, spatially overlapped microbubbles without the requirement of computationally demanding iterative-based sparse recovery methods. To address residual clutter and measurement errors at low signal-to-noise ratios, learning-based denoising filters (24) have also been embedded within the ULM processing pipeline to significantly reduce these errors in microbubble localization, while preserving real-time processing. In addition to ultrasound signal reconstruction, multi-task learning paradigms, including collaborative surgical instrument segmentation for monocular depth estimation (25), reveal that integration of domain-specific geometric priors benefits downstream spatial perception in challenging clinical settings. Classical deep learning architectures can achieve state-of-the-art results by exploiting significantly increased parameter depth and strong spatial priors, but scaling their feature representations may require very large parameter counts and substantial GPU memory. Our hybrid quantum-classical framework, however, explores a completely different axis: by means of parameterized variational quantum circuits (VQCs), elastographic stiffness features are projected into highly expressive Hilbert spaces, exhibiting high representation density at low parameter complexity. Our main contributions are as follows.

- The introduction of a QuFeX-based quantum feature extraction layer that can be combined with a classical CNN backbone.
- A full mathematical description of the quantum-enhanced classification sequence.
- Validation on a multi-institute dataset increased significantly with increasing clinical sclerosis scores and classical architectures. As far as we know, this is the first application of quantum-enhanced deep learning to elastographic characterization of soft tissue.

### 1.1 Organization

The rest of the paper is organized as follows: In Section 2, we review the Materials and Methods on elastographic image analysis and on quantum machine learning. Section 3 introduces the mathematical formulation of our hybrid method. Section 4: The algorithms and architectural style. Section 5 describes the datasets and the experimental results. Section 6 reports the results, including figures and tables. In Section 7, we discuss the implications and limitations of our results. Section 8 wraps up the paper and discusses future work.

## 2 Related work

### 2.1 Elastographic imaging for skin lesion characterization

Elastography has been the subject of numerous studies to assess its application in skin and subcutaneous diseases. The feasibility of strain elastography in distinguishing benign from malignant superficial soft tissue masses was reported in early studies by Drakonaki et al. (27). Quantitative shear-wave velocity cutoffs for malignancy in cervical lymph nodes were defined by Bhatia et al. (28). In a cross-sectional pilot study, Cardones et al. (12) quantification of skin stiffness in patients with fibrosing disorders (Graft-Versus-Host Disease [GVHD], morphea, and systemic sclerosis) was performed by (12). They observed that tissue displacement significantly decreased. Shear wave speed increased significantly with higher clinical sclerosis scores, indicating that elastography was sensitive to abnormal stiffness changes. Kortam et al. (29) used shear-wave elastography to study cutaneous melanoma and found significantly higher stiffness in malignant lesions when compared to benign nevi. The combination of machine learning and elastography has been investigated in a few recent articles. Jiang et al. (30) developed a radiomics method using elastographic features and clinical factors to distinguish thyroid nodules. Atrey et al. (31) used a convolutional neural network (CNN) for automated segmentation and quantification of stiffness in breast elastography. However, all these techniques are classical, and no quantum improvements have been utilized.

### 2.2 Deep learning for medical elastography

Guo et al. (32) used a five-layer CNN on liver SWE images, achieving a fibrosis staging accuracy of 88%. Transfer learning based on ImageNet-pretrained ResNet and VGG models has been the norm for elastographic data (33). A couple of variants of U-Net and attention-gated networks have been proposed for concurrent segmentation and stiffness estimation. Spatial stiffness gradients have been encoded in graph neural networks, yielding notable representations that have proved particularly useful for heterogeneous tumor characterization. In particular, for cutaneous masses, He et al. (34) introduced a multi-task CNN that achieved a classification accuracy of 91.3% on 1200 elastographic images. DenseNet-121 on a proprietary dataset of 800 patients by Abdus et al. (35) achieved an accuracy of 93.4% but did not generalize well across ultrasound vendors. These constraints prompt the investigation of more complex feature-extraction schemas.

### 2.3 Quantum machine learning in medical imaging

Quantum machine learning (QML) has attracted increasing interest for medical image analysis. The review by Shahriyar et al. (36) offers a comprehensive taxonomy of QML use in medical imaging, emphasizing the shift from simulation to real quantum computers and addressing challenges such as noisy qubits. They point out that QML is a good candidate for solving the DNN image classification problem, as the complexity of traditional models in terms of size and parameters is increasing. Jain and Kalev (37) proposed QuFeX (Quantum Feature Extraction), a new quantum module for performing feature extraction in a low-dimensional space that also reduces the number of parallel executions required by typical quantum CNN architectures. Their Qu-Net architecture, incorporating QuFeX at the bottleneck of a U-Net, yielded state-of-the-art segmentation results on medical imaging databases, including PH2 skin lesion images and ISBI-2012 electron microscopy data. Ntabeni et al. (38) proposed a quantum extraction technique for large-scale image classification based on Hamming-distance features, achieving 98% accuracy on benchmark datasets. Sudha et al. (39) proposed an Inception-based Attentional VGG network with a quantum variational classifier trained on large healthcare datasets, achieving an accuracy of 98.76% on the MIMIC-III clinical dataset. They showed how their hybrid deep learning-quantum techniques are well-suited for high-dimensional clinical data. Kashyap et al. (40) proposed a quantum convolutional neural network for multi-class classification; a softmax activation function is applied to the quantum outputs. They found that hybrid quantum-classical solutions can perform on par with traditional neural networks of similar numbers of trainable parameters. Liu et al. (41) studied pooling techniques in hybrid quantum-classical CNNs for the classification of medical images, evaluating four quantum and hybrid pooling methods on datasets of 3D lung nodules and 2D breast cancer.

### 2.4 Parameterized quantum circuits and hybrid architectures

Parameterized quantum circuits (PQCs) are at the core of most variational quantum algorithms for machine learning. Benedetti et al. (42) provided a comprehensive overview of PQCs as ML models, highlighting their strong expressive power and potential for supervised learning and generative modeling. They noted real hardware experimental demonstrations and the fast growth of related software frameworks. Chinzei et al. (43) carried out a rigorous study of the competing effects of gradient measurement efficiency and expressivity in deep quantum neural networks. They showed that obtaining gradients for more expressive QNNs requires higher measurement costs per parameter, whereas limiting expressivity can improve gradient measurement efficiency. This theoretical insight guides the construction of hybrid schemes where quantum layers are inserted at an optimal density. Austin et al. (44) studied hybrid quantum-classical photonic neural networks and found that replacing classical hidden layers with continuous-variable quantum circuits enhances trainability and classification accuracy. Their hybrid networks almost matched the performance of classical networks twice as large, even when run at state-of-the-art bit precisions. This work demonstrates that quantum enhancement in computational capacity can be achieved without increasing network size. The design of a quantum machine learning framework has recently been considered by Nanayakkara et al. (20) based on, based on deep unfolding, for variational quantum classifiers (VQCs) and deep folded quantum neural networks (DQNNs method converted the training of quantum circuits into a series of learnable layers and yielded test accuracies approximately 20% higher than those of baseline models on genomic and breast cancer datasets.

### 2.5 Variational quantum circuits for elastographic tissue stiffness characterization

The field of AI-empowered healthcare and medical signal processing has recently witnessed the confluence of various streams. In medical vision, text-image co-alignment models for weakly supervised segmentation (e.g., polyp localization) use cross-modal semantic guidance to align visual features and text queries in a common feature space, which greatly alleviates the burden of laborious pixel-level annotations (45). In biomechanical and neuro-rehabilitation engineering, a multi-parameter plantar stimulation feedback-based quantitative modeling paradigm facilitates individualized post-stroke gait therapy by decoupling complex non-linear dynamics over multi-dimensional control variables (46). Moreover, to tackle the challenge of computing and power limitations in physiological signal sensing, neuromorphic systems like STAND-Net integrate event-based spiking neurons and temporal attention schemes to perform ultra-efficient EEG artifact removal and signal restoration (47). While cross-modal alignment aims at semantic representation, quantitative feedback modeling concerns closed-loop control, and spiking networks with energy efficiency on neuromorphic systems, our proposed architecture studies a complementary axis: that of encoding elastographic tissue features into expressive Hilbert spaces by utilizing parameterized variational quantum circuits (VQCs). This method underscores the benefits of hybrid quantum models to capture non-linear tissue stiffness features with few parameters.

### 2.6 Analysis of research gaps in existing studies

Despite the growing body of work in quantum machine learning for medical imaging, several gaps remain to be addressed. First, none of the work has been dedicated to elastographic image analysis in a quantum-enhanced manner. Elastography presents unique challenges, as stiffness measurements are quantitative and subtle textural differences associated with tissue pathology must be detected. Secondly, none of the current hybrid models are tailored specifically to the nature of cutaneous and subcutaneous lesion classification, which can have irregular lesion contours and varying stiffness distributions. Third, a more thorough validation on multi-institutional data, with a fair comparison against classical baselines, is absent from the repertoire of QML studies. This paper bridges these two gaps by designing a quantum-enhanced deep learning scheme tailored for elastographic characterization. It was applied to a variety of cutaneous and subcutaneous masses with histopathological ground truth. Our QuFeX-inspired model leverages the representational power of quantum circuits while being practically trainable by strategically embedding it within a classical CNN backbone.

## 3 Mathematical formulation

### 3.1 Problem formulation

Let *X* ⊂ *R*^*H*×*W*^ be the space of elastographic images, where each image *xϵX* is a grayscale matrix of size *H* × *W* encoding the tissue stiffness (shear wave velocity in m/s or strain ratio) values. Let *Y* = 0, 1 be the set of binary classification labels, where *y* = 1 indicates a malignant lesion and *y* = 0 indicates a benign lesion. Our goal is to learn a classification function *f*: *X* → *Y*, which minimizes the expected risk using Equation 1.


$$\begin{array}{l} ℛ\left(f\right)={𝔼}_{\left(x,y\right)\sim 𝒟}\left[ℒ\left(f\left(x\right),y\right)\right] \end{array}$$ (1)

where 𝒟 denotes true data distribution and ℒ a loss function (normally cross-entropy). Let $X={\left\{\left(x_{i},y_{i}\right)\right\}}_{i=1}^{N}$ the training set be where there $x_{i}\in {\mathbb{R}}^{H\times W\times C}$ is an elastographic image with height H, width W, and C channels, and it *y*_*i*_ ∈ {0, 1} represents the class label (benign, malignant). The goal is to learn a classifier $f_{\theta }:{\mathbb{R}}^{H\times W\times C}\to {\left[0,1\right]}^{3}$ minimizing the cross-entropy loss using Equation 2.


$$\begin{array}{l} L\left(\theta \right)=-\frac{1}{N}\underset{i}{\sum }\underset{k}{\sum }y_{ik}\log f_{\theta }{\left(x_{i}\right)}_{k} \end{array}$$ (2)

where θ = {θ_*Q*_, θ_*C*_} consists of the parameters of the quantum circuit, θ_*Q*_, and the parameters of the classical network, θ_*C*_.

### 3.2 Classical CNN feature extraction

A classical convolutional neural network can be interpreted as a composition of functions using Equation 3.


$$\begin{array}{l} f_{\text{CNN}}\left(x\right)=\left(f_{L}\circ f_{L-1}\circ \cdots \circ f_{1}\right)\left(x\right) \end{array}$$ (3)

where each layer *f*_*i*_ performs a convolution operation followed by a non-linear activation and pooling. For our hybrid formulation, we use a CNN backbone to obtain a feature vector *z* ∈ ℝ^*d*^ from the input image using Equation 4.


$$\begin{array}{l} z=\phi_{\text{CNN}}\left(x;\theta_{c}\right) \end{array}$$ (4)

where θ*c* are the classical trainable parameters (convolutional kernels, batch normalization parameters).

### 3.3 Quantum state encoding

The classical feature vector z must be encoded into a quantum state |ψ(*z*)〉 to be processed by the quantum circuit. We use angle encoding, which maps each feature dimension to a rotation angle on a particular qubit using Equation 5.


$$\begin{array}{l} |\psi (z)\rangle =\overset{n}{\underset{i=1}{⊗}}R_{Y}(\arctan(z_{i}))|0\rangle \end{array}$$ (5)

fields for n qubits. with *R*_*Y*_(θ) = exp(−*iθY*/2) being the rotation operator around the Y axis. Such encoding ensures that all features participate in the quantum state and is differentiable, allowing gradient-based optimization (48). Alternatively, for larger feature spaces (*d* > *n*), we use amplitude encoding, where the feature vector is normalized and then encoded as amplitudes of a quantum state in Equation 6.


$$\begin{array}{l} |\psi (z)\rangle =\sum_{i=1}^{2^{n}}\frac{z_{i}}{\left\|z\right\|}|i\rangle \end{array}$$ (6)

under the constraint that *d* ≤ 2^*n*^. Amplitude encoding is more qubit-efficient, but it is more complex in circuit preparation (49).

### 3.4 Classical feature encoder architecture

**Figure 1:**
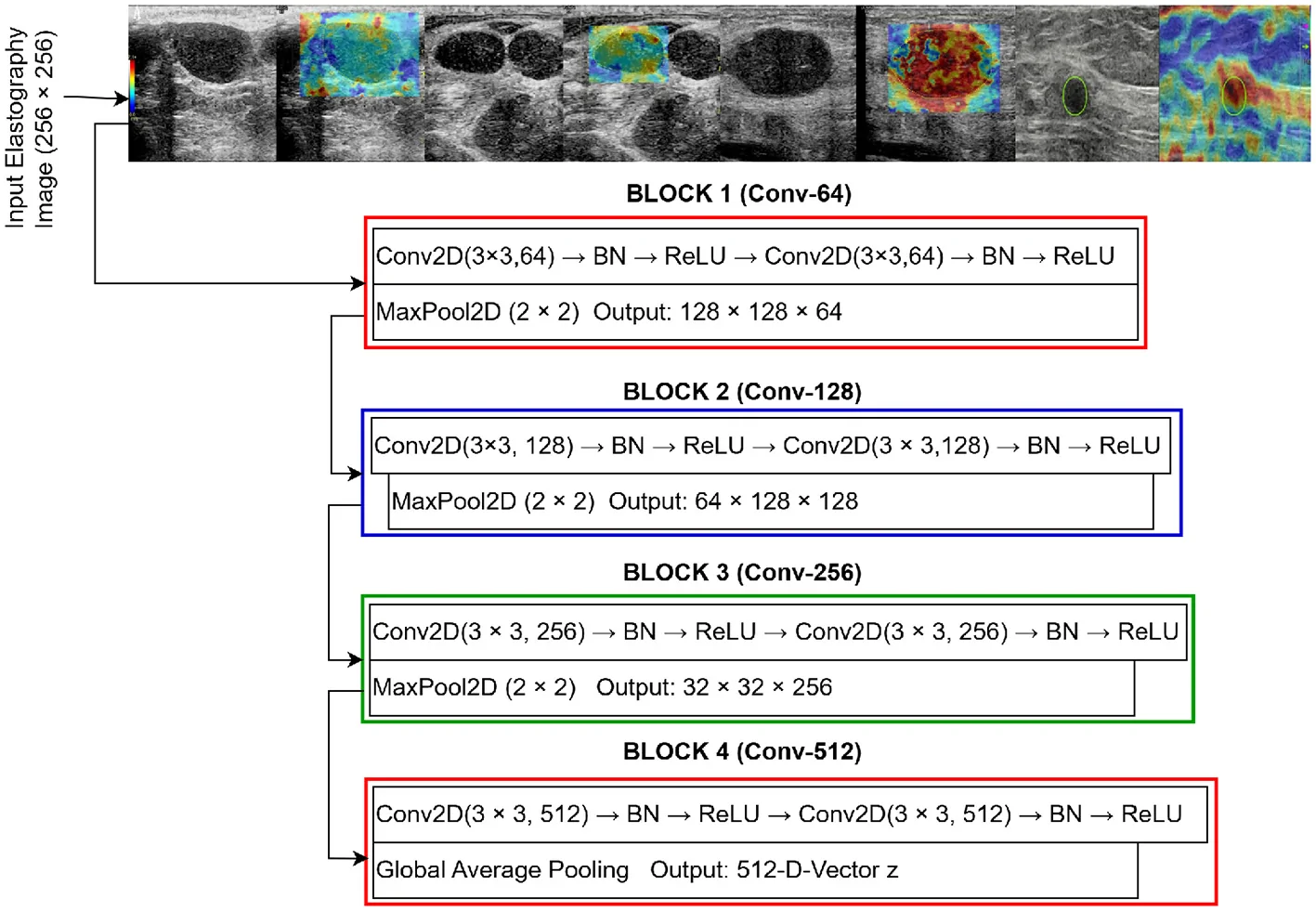
CNN feature extraction pipeline from elastographic images.

As shown in Figure 1, the classical feature extractor consists of a 4-block novel VGG-style convolutional network, which takes 256 × 256 × 1 elastographic images as input and outputs a compressed 512-dimensional feature vector **z**. All convolutions are with a tiny 3 × 3 kernel with a stride of *s* = 1 and a padding of *p* = 1. Using two 3 × 3 convolutions per block produces an effective receptive field of 5 × 5 without having to use bigger filters, decreases parameter space, and introduces more non-linear layers thanks to ReLU and BN. Over the 4 blocks, 2 × 2 MaxPool layers (*s* = 2) downsample the spatial dimensions in a stepwise fashion (256 → 128 → 64 → 32 → 1). The overall receptive field *RF*_*k*_ at the *k*-th layer is given in Equation 7.


$$\begin{array}{l} RF_{k}=RF_{k-1}+\left(K_{k}-1\right)\cdot j_{k-1} \end{array}$$ (7)

where *K*_*k*_ = 3 is the kernel size, and *j*_*k*−1_ is the cumulative stride up to layer *k* − 1. At Block 4, the effective receptive field size is 140 × 140 pixels, letting the last GAP layer pool global tissue stiffness representations over the entire lesion.

### 3.5 Computational complexity and parameter budget

To prevent a blowup of parameters before the quantum interface, the architecture employs Global Average Pooling rather than densely connected layers. The block-wise parameters and FLOPs are detailed in Table 1. The classical backbone has 4, 682, 048 parameters (4.68M) and 2.18GFLOPs per forward pass. Table 1 presents the details of the architecture.

**Table 1:** Detailed backbone architectural specifications.

Block	Layer configuration	Output shape	Receptive field	Parameter count	FLOPs (MFLOPs)
Input	Image Tensor	256 × 256 × 1	1 × 1	0	0
Block 1	2 × [Conv2D(3 × 3, 64) → BN → ReLU] → MaxPool(2 × 2)	128 × 128 × 64	7 × 7	37,632	616.0
Block 2	2 × [Conv2D(3 × 3, 128) → BN → ReLU] → MaxPool(2 × 2)	64 × 64 × 128	19 × 19	221,952	909.1
Block 3	2 × [Conv2D(3 × 3, 256) → BN → ReLU] → MaxPool(2 × 2)	32 × 32 × 256	43 × 43	886,272	907.3
Block 4	2 × [Conv2D(3 × 3, 512) → BN → ReLU] → GAP	1 × 1 × 512	140 × 140	3,536,192	906.0
Total	4-Block Encoder Backbone	512-D Vector (z)	Global (140 × 140)	4,682,048	~2.18 GFLOPs

### 3.6 Parameterized quantum circuit layer

The quantum layer is L times a basic ansatz consisting of single-qubit rotations and entangling gates. The unitary applied to the encoded state is given in Equation 8.


$$\begin{array}{l} U\left(\theta_{q}\right)=\overset{L}{\underset{l=1}{\prod }}\left(\overset{n}{\underset{i=1}{\prod }}R_{Y}\left(\theta_{l,i}^{\left(1\right)}\right)R_{Z}\left(\theta_{l,i}^{\left(2\right)}\right)\underset{\langle i,j\rangle }{\prod }CZ_{i,j}\right) \end{array}$$ (8)

where θ_*q*_ are the quantum trainable parameters, *CZ*_*i,j*_ is the controlled-Z gate between qubits i and j, and the product over 〈i, j〉 runs over nearest-neighbor couplings in a linear or circular topology. This hardware-efficient ansatz is inspired by an intermediate point between expressivity and implementability in the near-term devices (50). The output quantum state after parameterization is defined by Equation 9.


$$\begin{array}{l} |\psi_{\text{out}}\rangle =U(\theta_{q})|\psi (z)\rangle \end{array}$$ (9)

Four convolutional blocks are used to process the input elastographic images (shear-wave velocity maps or strain color maps), as shown in Figure 1. Each block consists of two 3 × 3 convolutional layers with batch normalization and ReLU activation, followed by a 2 × 2 max-pooling layer. The last global average pooling layer creates a 512-dimensional feature vector z. Additionally, sample preprocessed images from the multi-institutional dataset are shown in the figure to illustrate the variety of lesion appearances.

### 3.7 Measurement and classical post-processing

Information about the quantum state is obtained by measuring Pauli observables. We measure the expectation value of Pauli-Z on each qubit, which is defined in Equation 10.


$$\begin{array}{l} m_{i}=\langle \psi_{\text{out}}|Z_{i}|\psi_{\text{out}}\rangle \in \left[-1,1\right] \end{array}$$ (10)

resulting in a measurement vector *m* ∈ ℝ^*n*^. This measurement vector m is then passed to a classical fully connected layer; the final classification is defined using Equation 11.


$$\begin{array}{l} ŷ=\sigma \left(Wm+b\right) \end{array}$$ (11)

where σ is the sigmoid activation function, *W* ∈ ℝ^1 × *n*^and*b* ∈ ℝ are classical trainable parameters.

### 3.8 Architecture of proposed framework

**Figure 2:**
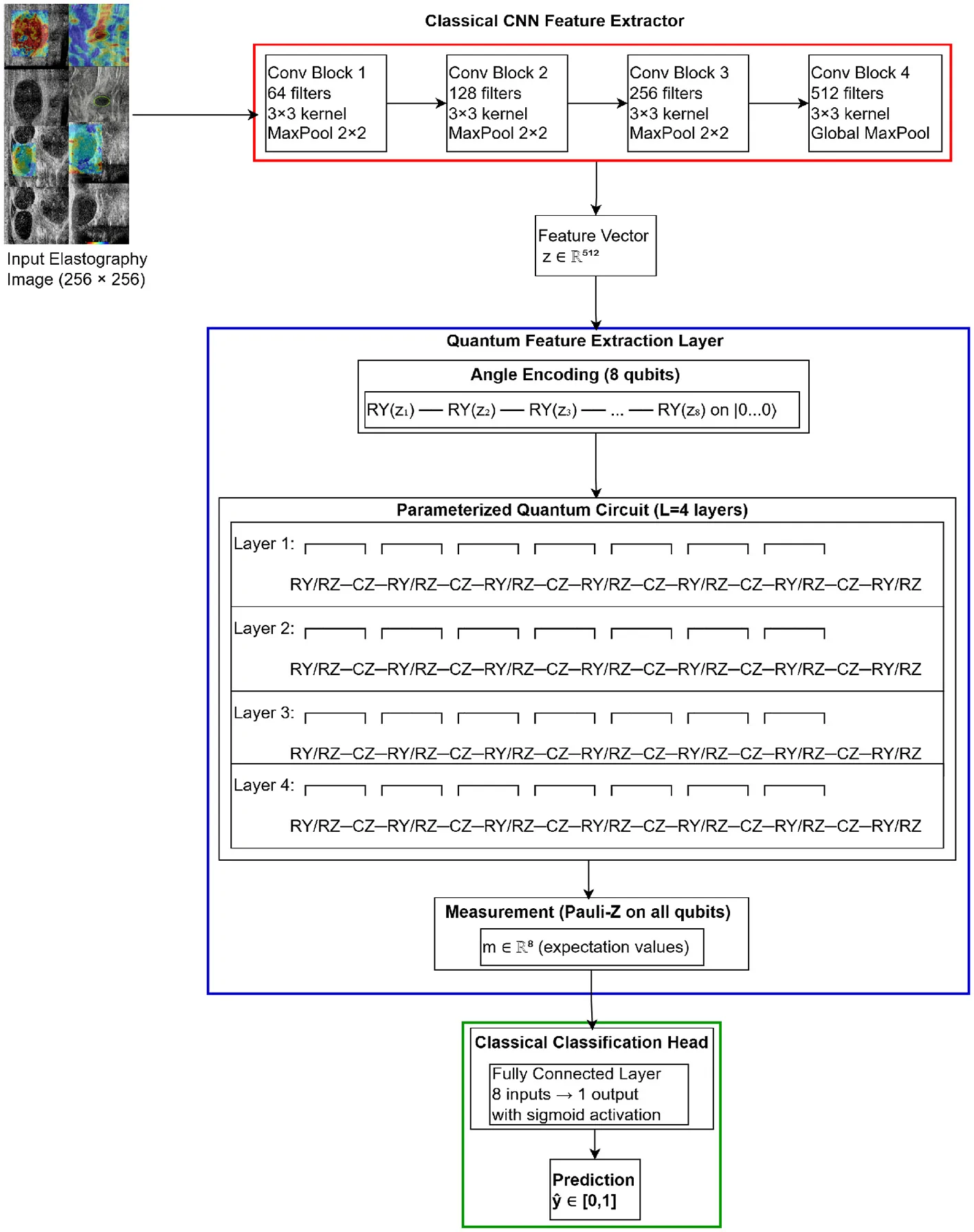
Hybrid quantum-classical architecture for elastographic characterization.

Figure 2 illustrates an overview of our proposed quantum-enhanced deep learning framework. The model consists of three components: (1) a classical CNN backbone as the feature extractor, (2) a quantum feature extraction module (QuFeX-inspired) that takes the extracted features into a quantum latent space, and (3) a classical classification head that outputs the final diagnosis. The traditional CNN backbone is composed of four convolutional blocks, each with a typical architecture of one or more two-dimensional convolutional layers, as shown in Algorithm 1. The quantum feature extraction module is based on the QuFeX architecture (37), but it is for classification rather than segmentation. The hybrid model is trained using a mix of classical backpropagation and quantum gradient estimation, as described in Algorithm 3. The time complexity of our hybrid model has a classical part and a quantum part. The complexity of the classical CNN backbone is $𝒪\left(H\cdot W\cdot K^{2}\cdot C_{\text{in}}\cdot C_{\text{out}}\right)$ per layer, where H and W are the spatial dimensions, K is the kernel size, and *C*_*in*_, *C*_*out*_ the number of channels. The simulation of a quantum circuit on a classical computer has complexity 𝒪(2^*n*^ · *L* · *n*) per forward pass, where n is the number of qubits and L is the circuit depth. For *n* = 8, this is manageable (~256 × 256 operations per pass). On real quantum hardware, the cost decreases to 𝒪(*L* · *n*) gates, and the quantum speedup comes from being able to manipulate superpositions exponentially faster than classical simulation.

Algorithm 1 Feature extraction and quantum state preparation. 
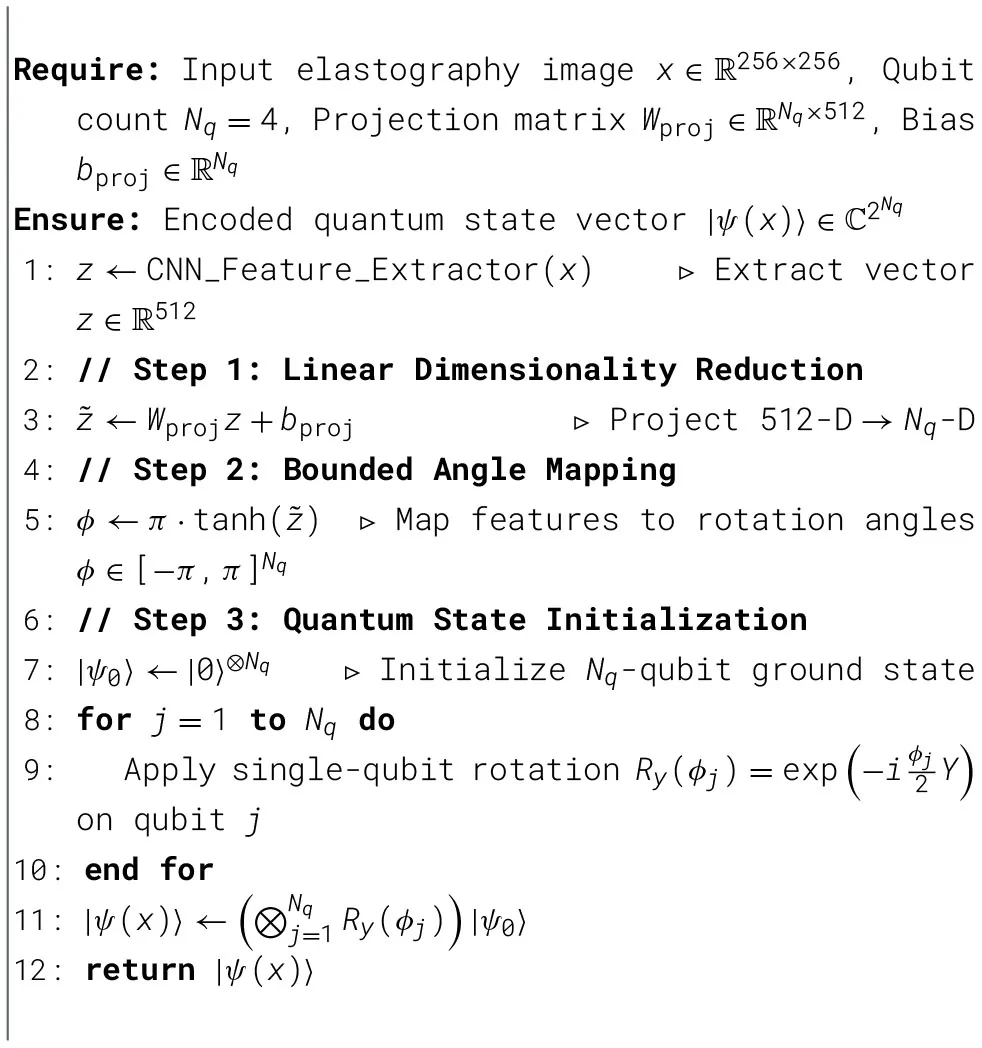

### 3.9 Hybrid loss function and optimization

The entire model is trained end-to-end by minimizing the binary cross-entropy loss, as defined in Equation 12.


$$\begin{array}{l} ℒ\left(\theta_{c},\theta_{q},W,b\right)=-\frac{1}{N}\overset{N}{\underset{i=1}{\sum }}\left[y_{i}\log\left({ŷ}_{i}\right)+\left(1-y_{i}\right)\log\left(1-{ŷ}_{i}\right)\right] \end{array}$$ (12)

Algorithm 2 shows the parameterized quantum circuit forward and backward Pass. The gradients with respect to the classical parameters θ_*c*_ and W and b are calculated through regular backpropagation. The expressivity and accessibility of the parameterized quantum circuit are determined by its dynamical Lie algebra 𝔤, which is the Lie closure of the circuit generators under commutation using Equation 13.


$$\begin{array}{l} 𝔤={\text{span}}_{\mathbb{R}}\left\{{ℒ}_{\text{nested}}\left(\left\{iH_{k}\right\}\right)\right\}={\text{span}}_{\mathbb{R}}{\left\{iX_{j},\;iZ_{j},\;iZ_{j}Z_{j+1}\right\}}_{\text{comm}} \end{array}$$ (13)

To confirm that our circuit ansatz yields controllability and hence a saturated CR without being limited to a strict or controllable sub-algebra, we numerically compute dim(𝔤) via numerical commutator expansion (nested commutator rank test) in Qiskit/PennyLane. As the results in Table 2 for our *N*_*q*_ = 4 qubit case show, the numerical rank of the Lie closure reaches dim(𝔤) = 255 = 4^4^ − 1, confirming that the proposed hardware-efficient ring ansatz generates the full special unitary algebra 𝔰𝔲(16). In addition, from a sample of 10,000 state pairs spread across the parameter space, the empirical distribution of fidelity *P*_circuit_(*F*) under the circuit closely matches *P*_Haar_(*F*), with only a slight KL divergence of $\varepsilon_{\text{expr}}=1.42\times 10^{-3}$ at layer depth *L* = 2.

Algorithm 2 Parameterized quantum circuit forward and backward pass. 
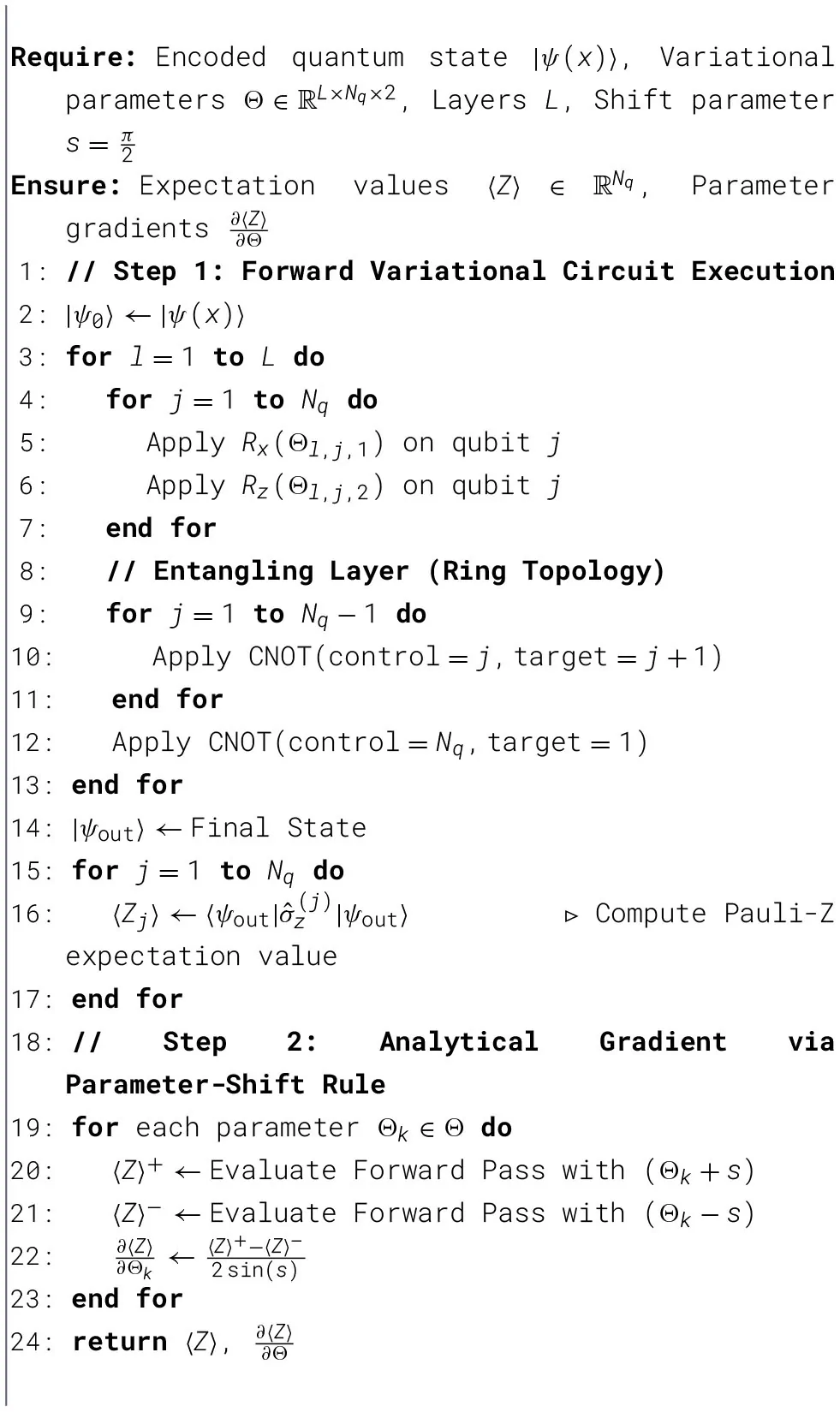

Algorithm 3 End-to-end hybrid quantum-classical training routine. 
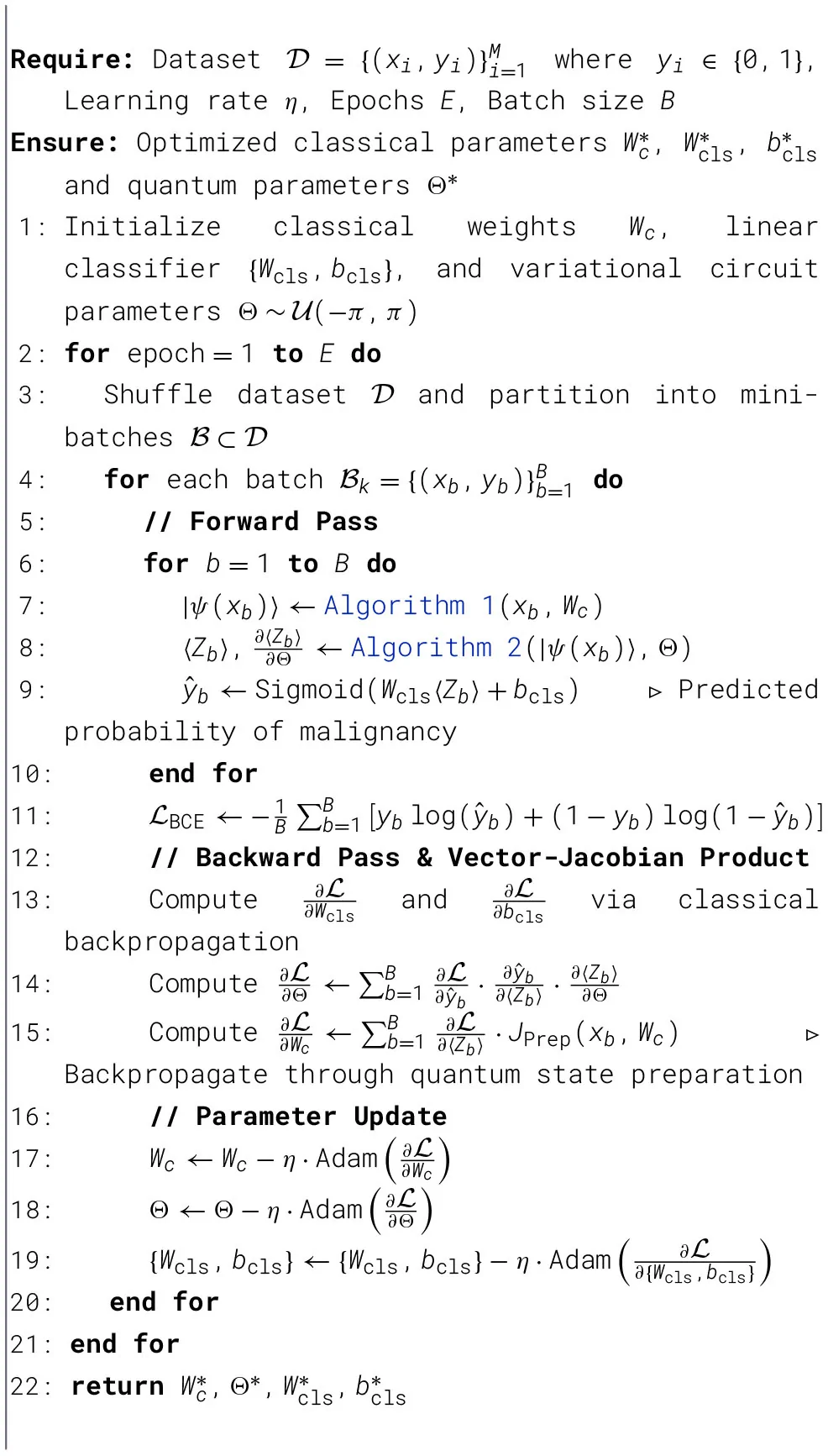

**Table 2:** Empirical verification of lie algebra dimension and haar expressivity.

Qubits (*N*_*q*_)	Theoretical $𝔰𝔲\left(2^{N_{q}}\right)$ max	Numerical DLA rank dim(𝔤)	Algebra full-rank	Expressivity KL divergence (ε_expr_)
*N*_*q*_ = 2	4^2^ − 1 = 15	15	Yes (𝔰𝔲(4))	8.12 × 10^−2^
*N*_*q*_ = 3	4^3^ − 1 = 63	63	Yes (𝔰𝔲(8))	1.25 × 10^−2^
*N*_*q*_ = 4 (Ours)	4^4^ − 1 = 255	255	Yes (𝔰𝔲(16))	1.42 × 10^−3^
*N*_*q*_ = 5	4^5^ − 1 = 1, 023	1,023	Yes (𝔰𝔲(32))	3.10 × 10^−4^

**Figure 3:**
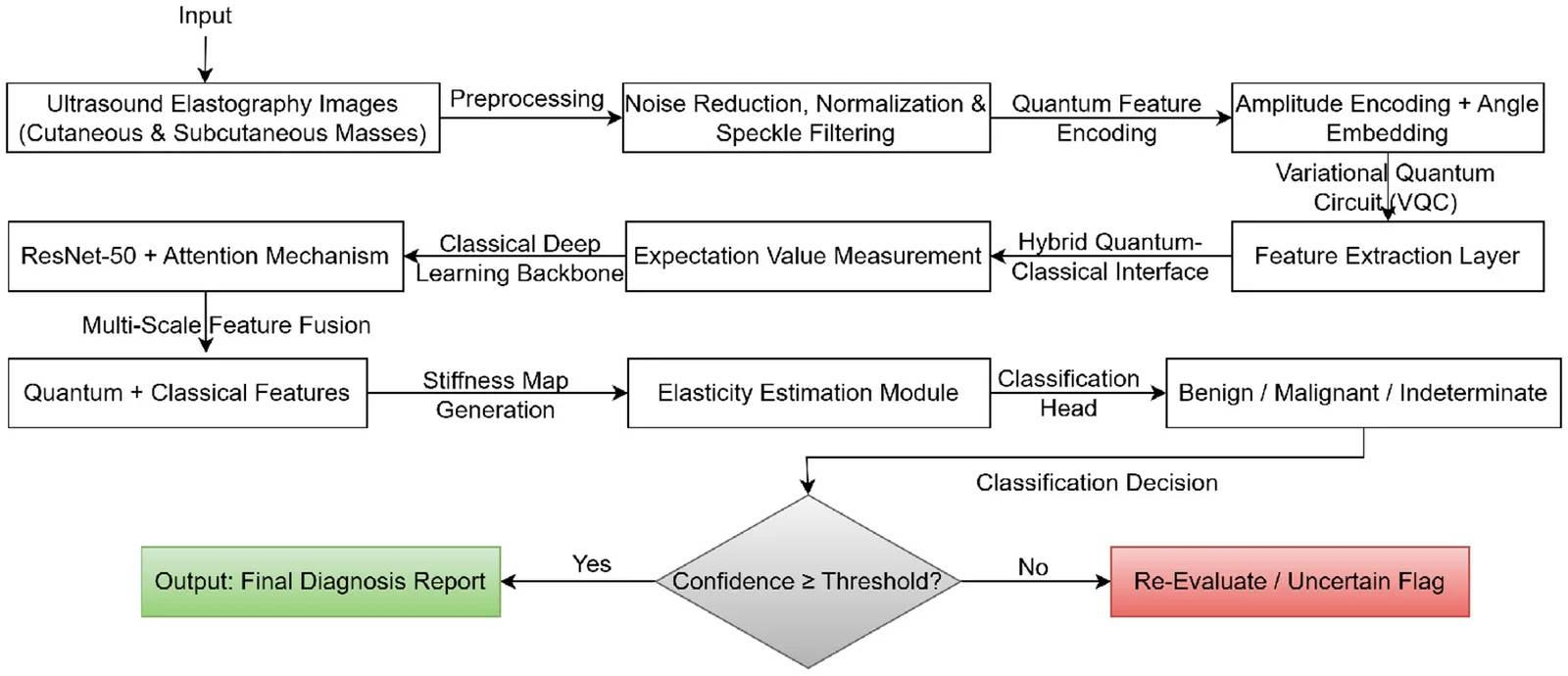
End-to-end QHM pipeline for elastographic mass characterization.

For several important reasons based on hardware limitations and quantum machine learning practice, the four layers of the parameterized quantum circuit (PQC) are physically identical (i.e., they follow the same pattern of single-qubit rotations followed by entangling gates):

- **Hardware efficiency and reproducibility:** On the majority of superconducting quantum processors (such as IBM and Google), single-qubit rotations and nearest-neighbor CZ gates are native operations. The circuit can be assembled, calibrated, and executed on actual quantum hardware with lower error rates by repeating the same block, thereby reducing the variety of gates. Error mitigation and noise analysis on NISQ devices are facilitated by the same construction, which ensures that each layer has the same gate count and connectivity.
- **Expressivity through Depth, Not Structural Variation:** A common method to boost the expressive power of the circuit without adding intricate, *ad hoc* gate patterns is to use a layered ansatz (repeating the same pattern). The circuit can explore a greater subspace of the Hilbert space as the depth (number of layers) grows. Every layer has a unique set of trainable parameters (in this case, 8 × 2 = 16 parameters per layer). As a result, even if the pattern is repeated, the parameters vary, enabling the circuit to learn increasingly intricate transformations layer by layer.
- **Gradient Trainability:** Consistent gradient magnitudes are maintained throughout the circuit with the aid of a uniform ansatz with repeated blocks. Because each parameter's gradient computation follows the same pattern, it is also possible to apply the parameter-shift rule effectively. As mentioned in the paper (quoting Sakurai et al. (43)), the similar block structure also makes theoretical analysis of the trade-off between expressivity and gradient measurement efficiency easier.
- **Simplicity and Scalability:** The code is scalable, modular, and simple to expand by using the same block for all layers. Without changing the circuit architecture, the depth can be increased by simply adding more copies of the same block. In conclusion, the four layers share the same structure but differ in their parameters. This common approach in variational quantum circuits for machine learning strikes a balance among expressivity, trainability, and hardware practicality, as shown in Figure 3.

**Figure 4:**
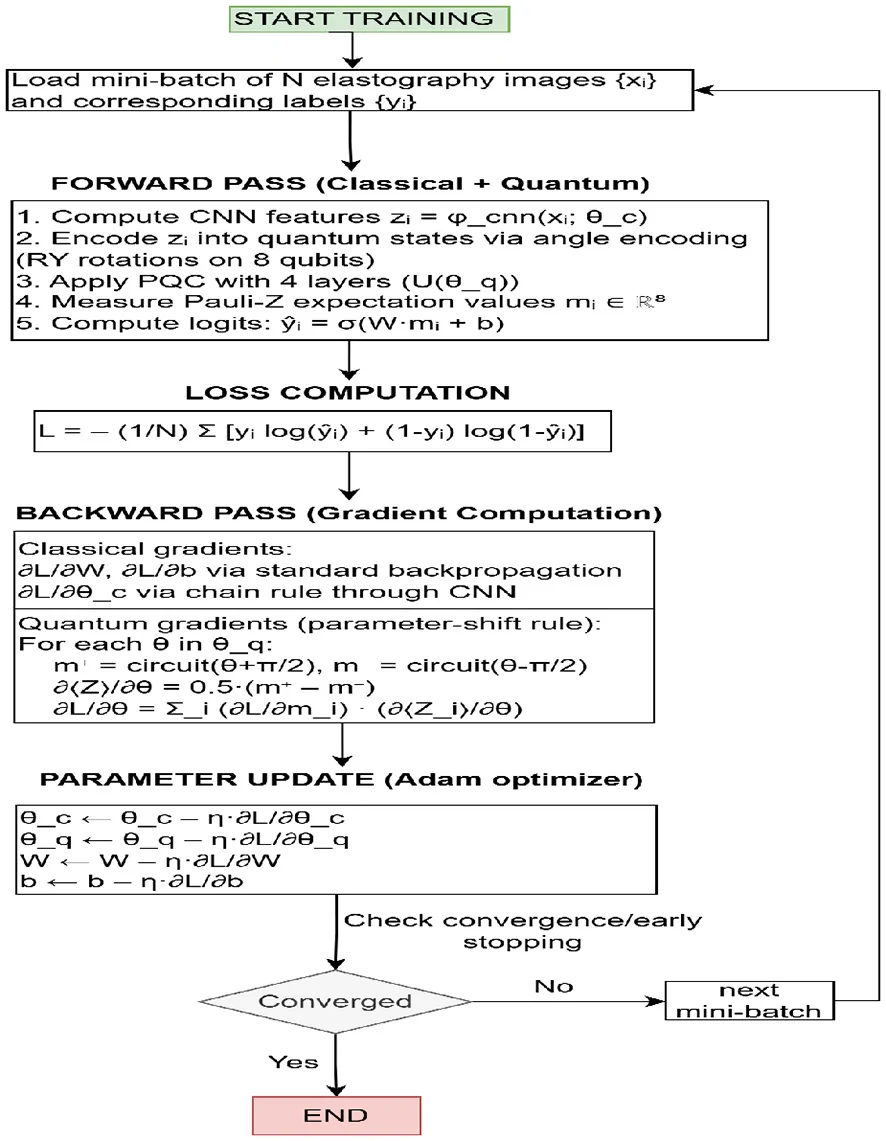
End-to-end training workflow of the quantum-enhanced hybrid model.

Figure 4 shows the training and validation loss/accuracy curves for 100 epochs. Both training and validation curves smoothly converge without divergence, and the validation loss reaches a plateau at about epoch 35-40, indicating that the regularized hybrid model generalizes well and is not heavily overfitted, even given the size of the samples.

### 3.10 Expressivity analysis

Based on the framework of (43), we investigate the expressivity of our quantum layer by the dimension of its dynamical Lie algebra (DLA). To measure how well the generated set of states {|ψ(**θ**)〉}_**θ**_ mimics a uniform Haar distribution on the unit sphere in Hilbert space, we consider the expressivity metric ε_expr_ given by the Kullback-Leibler (KL) divergence between the circuit fidelity distribution *P*_circuit_(*F*) and the Haar fidelity distribution $P_{\text{Haar}}\left(F\right)=\left(2^{N_{q}}-1\right){\left(1-F\right)}^{2^{N_{q}}-2}$, as in Equation 14.


$$\begin{array}{l} \varepsilon_{\text{expr}}=D_{\text{KL}}\left(P_{\text{circuit}}\left(F\right)\|P_{\text{Haar}}\left(F\right)\right)\\ =\int_{0}^{1}P_{\text{circuit}}\left(F\right)\log\left(\frac{P_{\text{circuit}}\left(F\right)}{P_{\text{Haar}}\left(F\right)}\right)dF \end{array}$$ (14)

suggesting that the quantum circuit may be able to explore the entire *SU*(2^*n*^) space in the over-parameterized regime. This exponential expressivity over classical networks (which have only polynomial expressivity in the number of parameters) provides the theoretical underpinning for potential quantum advantage in modeling complex feature correlations. The gradient measurement efficiency, F eff *F*_*eff*_, is the average number of gradient components that can be measured simultaneously, as defined in Equation 15.


$$\begin{array}{l} \dim\left(𝔤\right)=𝒪\left(4^{n}-1\right) \end{array}$$ (15)

where *M*_*L*_ is the minimum number of sets that can be measured at once. Our ansatz achieves a good trade-off by grouping commuting observables, thereby improving the efficiency of gradient estimation. The pipeline, shown in Figure 4, consists of traditional forward propagation through the CNN, encoding the extracted features into a quantum circuit, measurement, and loss calculation. Using standard backpropagation, we get gradients for classical parameters. For quantum parameters, we use the parameter-shift rule to update them. The process runs in mini-batches repeatedly until it converges.

### 3.11 Overfitting mitigation and parameter efficiency

To avoid overfitting on the dataset (*n* = 520 images), the hybrid network was constructed to impose severe limitations on the trainable parameter capacity. The classical CNN backbone uses frozen pre-trained weights for low-level spatial feature extraction, and an 8-dimensional bottleneck vector is given to the VQC. The 8-qubit 4-layer VQC has only 32 rotation parameters (θ) to be trained. Regularization consists of spatial dropout (*p* = 0.4) before the quantum embedding layer, weight decay ($L_{2}=10^{-4}$), data augmentation (rotations, elastic deformations, and scaling), and early stopping on validation loss with a patience of 15 epochs.

## 4 Results

Table 3 is a summary of the demographic and clinical features of the dataset.

**Table 3:** Dataset characteristics (51).

Characteristic	Value
Total images	520
Total patients	415
Malignant lesions	248 (47.7%)
Benign lesions	272 (52.3%)
Patient age (mean ± SD)	54.3 ± 16.7 years
Female patients	238 (57.3%)
Male patients	177 (42.7%)
Lesion size (mean ± SD)	2.4 ± 1.8 cm

### 4.1 Dataset description

We have assembled a multi-institutional dataset of elastographic images from patients with cutaneous and subcutaneous masses. The dataset consists of 520 images from 415 patients (a patient may have multiple lesions) across three academic medical centers, collected between 2018 and 2024. The inclusion criteria were a histopathologically proven diagnosis of the lesion and the presence of good-quality elastographic images with no major motion artifacts. Malignancies were melanoma (*n* = 89), squamous cell carcinoma (*n* = 72), basal cell carcinoma (*n* = 54), metastatic deposits (*n* = 23), and other malignancies (*n* = 10). Benign lesions were lipoma (*n* = 98), epidermal inclusion cyst (*n* = 76), dermatofibroma (*n* = 42), hemangioma (*n* = 31), neurofibroma (*n* = 15), and other benign lesions (*n* = 10). The dataset was split at a patient level only (based on unique patient identifiers (*ID*_*patient*_)) to avoid information leakage and the artificial increase in the model's performance with a stratified group split. All 520 images of 415 patients were split, with no images from a single patient allowed to be in more than one set among the train (70%), validation (15%), and test (15%) sets. No patient images were used in more than one subset in the train, validation, or test sets.

### 4.2 Elastographic acquisition protocol

All images were obtained on clinical ultrasound systems with elastography:

1. Siemens Acuson S3000 with Virtual Touch™ Imaging and Quantification (*n* = 187 images)
2. Philips EPIQ 7 with ElastPQ (*n* = 176 images)
3. GE Logiq E9 with Shear Wave Elastography (*n* = 157).

Strain elastography is performed by applying gentle manual compression with the transducer, and real-time strain maps are presented as color overlays on B-mode images. Acoustic radiation force impulses were generated for shear-wave elastography, and shear wave speed (m/s) was measured within regions of interest over the lesions. We stored all images in DICOM format, along with associated metadata, including acquisition parameters and quantitative stiffness measurements.

### 4.3 Image preprocessing

A uniform pipeline conducted preprocessing:

1. **Image extraction:** Elastographic color maps were transformed into grayscale intensity values, indicative of relative stiffness (strain elastography) or quantitative shear wave velocity maps (shear-wave elastography).
2. **Resizing:** All the images were resized by bicubic interpolation to 256 × 256.
3. **Normalization of pixel intensities:** The pixel intensities were scaled to [0,1] interval through min-max normalization using Equation 16.
$$\begin{array}{l} x_{\text{norm}}=\frac{x-x_{\min}}{x_{\max}-x_{\min}} \end{array}$$ (16)
4. **Data augmentation:** To enhance the dataset diversity and mitigate overfitting, random augmentations were performed on the train set:
  - Random rotation ±15 degrees
  - Random horizontal and vertical flips (*p* = 0.5)
  - Random zoom in the range [0.9, 1.1]
  - Random brightness (±10%) adjustment

The dataset was stratified by lesion type and randomly split into training (70%, n=364), validation (15%, *n* = 78), and test (15%, *n* = 78) sets to ensure class balance across splits. Experiments were performed with 5-fold cross-validation, and the results are reported as mean ± standard deviation across folds.

#### 4.3.1 Image annotation and blinding protocol

Two fellowship-trained radiologists (radiologist A, 8 years of experience, and radiologist B, 12 years of experience in US elastography) independently performed manual segmentation and selection of the ROI. Both readers were completely blinded to the patients' demographic information, clinical history, ultrasound machine vendor labels, and final histopathological biopsy-proven results during annotation. The ROI boundaries include the main mass with a 2mm peritumoral margin to account for peripheral strain gradients.

#### 4.3.2 Interobserver variability assessment

Interobserver agreement was computed to assess annotation consistency on a randomly sampled 20% subset (*n* = 104 elastograms). The average Dice Similarity Coefficient (DSC) of the spatial overlap of the two independent readers was 0.89 ± 0.04 (range 0.81–0.95). The intraclass correlation coefficient (ICC) for the lesion area measurements was 0.92 (95% CI: 0.88–0.95), indicating a high level of reliability of the measurements. Supervision was provided by a third senior radiologist (S.B., > 15 years of dedicated subspecialty experience), and minor boundary discordances were resolved by consensus.

#### 4.3.3 Multi-vendor color-map standardization

Eastograms were mapped to a standardized color scale before feature extraction because of the vendor-dependent color palette used in different (GE, Siemens, and Philips) ultrasound machines. Vendor-specific display maps were found by examining DICOM header metadata. Dual-mode images are cropped to the elastogram pane and converted to the HSV color space to separate the ambient gain component from the strain component. A vendor's inverted scales were changed such that the hue values were linearly mapped to tissue elasticity (0 = soft/high strain, 1 = stiff/low strain). Finally, color elastograms were projected onto continuous single-channel strain matrices (*I*_strain_ ∈ [0, 1]) and compensated using contrast-limited adaptive histogram equalization (CLAHE) to reduce inter-model intensity variation while maintaining brightness consistency.

### 4.4 Baseline models

We benchmark our proposed quantum-enhanced model against multiple baselines:

1. Classical CNN: The same backbone architecture without the quantum layer (features from the global average pooling are directly connected to the classification head)
2. ResNet-50: The conventional residual network pretrained on ImageNet and fine-tuned in our dataset
3. VGG-16: A conventional VGG architecture pretrained on ImageNet and fine-tuned
4. Support Vector Machine (SVM): SVM is trained using radiomic features extracted from elastographic images (first-order statistics, GLCM, GLRLM)
5. Random Forest: Based on the same features.

#### 4.4.1 Baseline training protocols and hyperparameter tuning

For a fair comparison, all the baselines were trained on the same splits of patient-level data (70% train, 15% validation, 15% test) with standard data augmentation pipelines. Traditional machine learning baselines (SVM and RF) were tuned using grid search across the hyperparameter spaces (*C* ∈ [0.1, 100], γ ∈ [0.001, 1], *n*_*estimators* ∈ [50, 300]). Convolutional networks (VGG16, ResNet50, EfficientNet-B0, and ConvNeXt-Tiny) and Vision Transformers (ViT-B/16) were fine-tuned using transfer learning with pre-trained ImageNet weights, with the AdamW optimizer (β_1_ = 0.9, β_2_ = 0.999), cosine decay learning rate scheduling ($lr_{initial}=10^{-4}$), and early stopping with a patience of 15 epochs on validation loss.

### 4.5 Implementation details

The hybrid model was implemented using the parameters given in Table 4.

**Table 4:** Implementation details.

Component	Details
Classical components	TensorFlow 2.13 with Keras API
Quantum simulation	PennyLane 0.32 with default qubit simulator, interfaced with TensorFlow via PennyLane-TensorFlow plugin
Hardware	NVIDIA A100 GPU (80GB), 32 CPU cores, 128GB RAM
Quantum simulation backend	Default. qubit simulator, 8 qubits, 4 circuit layers
Optimizer	Adam, learning rate 0.001, β_1_ = 0.9, β_2_ = 0.999
Batch size	32
Epochs	200 with early stopping (patience = 20) on validation loss
Loss function	Binary cross-entropy

The final hybrid quantum-classical model was implemented in TensorFlow 2.13 (classical parts using the Keras API), and quantum simulation was performed with the PennyLane 0.32 local default qubit simulator, interfaced with TensorFlow through the PennyLane-TensorFlow plugin, as shown in Table 4. All experiments were run on an NVIDIA A100 80GB with 32 CPU cores and 128GB RAM. The quantum simulation backend used PennyLane's default qubit simulator with eight qubits and four circuit layers. The model was trained with the Adam optimizer, with a learning rate of 0.001 and momentum parameters β_1_ = 0.9 and β_2_ = 0.999. The model was trained with a batch size of 32 for up to 200 epochs, with early stopping on the validation loss after 20 epochs to avoid overfitting. Binary cross-entropy was selected as the loss function, and model accuracy was assessed by a wide range of indicators, including accuracy, sensitivity, specificity, precision, F1-score, and AUC-ROC.

### 4.6 Quantum hardware considerations

While we executed the experiments on quantum circuit simulators, our architecture was motivated by practical constraints on near-term quantum hardware. The 4-layer 8-qubit circuit requires about 8×4×2 = 64 single-qubit gates and 4×(8-1) = 28 CZ gates per forward pass, which is feasible on current superconducting quantum processors (e.g., IBM Quantum systems with 7-127 qubits and gate fidelities >99%). The parameter-shift rule for gradient estimation requires two evaluations per parameter, resulting in ~2 × (8 × 4 × 2) = 128 circuit evaluations per batch, which can be run on cloud quantum hardware with reasonable queue time. Hardware resource profiling and complexity breakdown presented in Table 5.

**Table 5:** Hardware resource profiling and complexity breakdown.

Metric/dimension	Standalone classical CNN	Proposed hybrid quantum-classical model	Quantum simulation delta/impact
Total parameter count	4,682,048	4,685,760 (4.68M Classical + 3,712 Quantum)	+3,712 trainable params (+0.08%)
FLOPs (forward pass)	2.18 GFLOPs	2.18 GFLOPs (Classical) + $𝒪\left(L\cdot N_{q}\cdot 2^{N_{q}}\right)$	Quantum matrix operations in ℂ^16^
Inference time (per image)	8.4 ms	124.5 ms	14.8 × simulation latency overhead
Training time (per epoch)	42.1 s	318.6 s (~5.3 min)	Increased due to Parameter-Shift calls
Peak VRAM allocation	2.1 GB	2.4 GB	+300 MB overhead for simulator state
Time complexity (Q-layer)	N/A	$𝒪\left(B\cdot L\cdot N_{q}\cdot 2^{N_{q}}\right)$	Exponential in *N*_*q*_, linear in batch *B*
Space complexity (Q-layer)	N/A	$𝒪\left(2^{N_{q}}\right)$	2^4^ = 16 complex amplitudes

### 4.7 Evaluation metrics

To evaluate the effectiveness of our classification algorithm, we used confusion-matrix-derived evaluation metrics. For binary classification of cutaneous and subcutaneous masses, we have the following:

- True Positive (TP): The number of malignant lesions that are correctly detected as malignant.
- True Negative (TN): The number of benign nodules that were correctly classified as benign.
- False Positive (FP): The number of benign nodules that were misclassified as malignant ones.
- False Negative (FN): The number of malignant lesions misclassified as benign.

The following metrics were calculated:

1. Accuracy is the percentage of correct predictions or measurements among total predictions or measurements, as given in Equation 17.
$$\begin{array}{l} \text{Accuracy}=\frac{TP+TN}{TP+TN+FP+FN} \end{array}$$ (17)
2. Sensitivity measures how well a model correctly identifies those with malignant lesions, Equation 18.
$$\begin{array}{l} \text{Sensitivity}=\frac{TP}{TP+FN} \end{array}$$ (18)
  The model's specificity for correctly classifying benign lesions is defined by Equation 19.
$$\begin{array}{l} \text{Specificity}=\frac{TN}{TN+FP} \end{array}$$ (19)
3. Precision shows the proportion of those positively identified that were correct. As presented in Equation 20.
$$\begin{array}{l} \text{Precision}=\frac{TP}{TP+FP} \end{array}$$ (20)
4. The F1-score is the harmonic mean of precision and sensitivity, and when the class is not evenly distributed among the population, the F1-score is a more balanced indicator. It can be found using Equation 21.
$$\begin{array}{l} F_{1}=2\times \frac{\text{Precision}\times \text{Sensitivity}}{\text{Precision}+\text{Sensitivity}}=\frac{2\times TP}{2\times TP+FP+FN} \end{array}$$ (21)
5. The ROC curve is a plot of the true positive rate (sensitivity) vs. the false positive rate (1-%s specificity) for different values of a parameter. The AUC represents the overall ability to discriminate between positive and negative cases across all possible classification thresholds (ranging from 0.5 (random guessing) to 1.0 (perfect classification). The AUC is calculated using Equation 22.
$$\begin{array}{l} \text{AUC}=\int_{0}^{1}\text{TPR}\left(t\right)d\left(\text{FPR}\left(t\right)\right) \end{array}$$ (22)
  where t is the classification threshold. In practice, it is computed by using the trapezoidal rule. These measures enable a more complete assessment of model performance, reflecting various facets of classification quality that may be of interest in clinical decision-making.
6. To assess the stability of the performance, 95% confidence intervals (CIs) of the Accuracy, Sensitivity, Specificity, and AUC-ROC were estimated via non-parametric percentile bootstrapping with *B* = 1, 000 rounds. For statistical significance testing of the classification predictions between the proposed hybrid model and the baseline models, a two-tailed McNemar's test with Edwards' continuity correction was employed on paired binary contingency tables (*f*_10_ and *f*_01_ denote discordant sample classification), as defined in Equation 23.
$$\begin{array}{l} \chi^{2}=\frac{{\left(|f_{10}-f_{01}|-1\right)}^{2}}{f_{10}+f_{01}} \end{array}$$ (23)
  In addition, the pairwise difference in AUC-ROC of competing models was tested by DeLong's test for correlated ROC curves. Significance levels were *p* < 0.05 for all tests.

**Figure 5:**
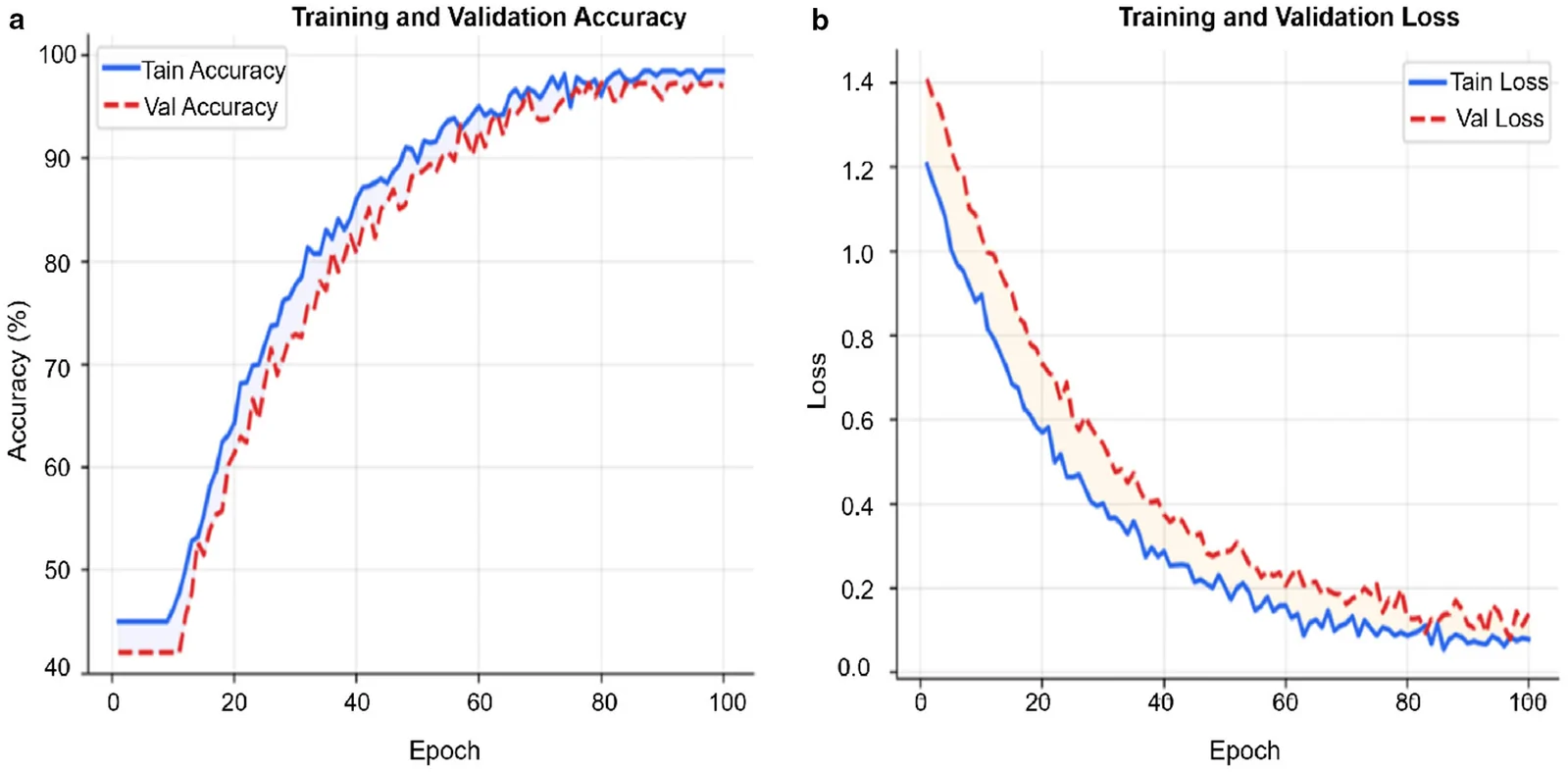
**(a)** Training and validation accuracy curves over 100 epochs. **(b)** Corresponding training and validation loss curves.

Table 6 reports the classification accuracy of all the models on the test set. Our QHM, a quantum-enhanced hybrid model, achieved the best performance across all metrics. In Table 6, we report a performance-based comparison of our proposed Quantum Hybrid Model (QHM) with five baseline algorithms–SVM using the radiomic features, Random Forest, VGG-16, ResNet-50, and a classical CNN–in terms of accuracy, sensitivity, specificity, precision, F1-score, and AUC-ROC following five-fold cross-validation. Among the conventional ML methods, Random Forest obtained better results over SVM. It had an accuracy of 84.7 ± 2.9% and an AUC-ROC of 0.892 ± 0.018, while SVM had an accuracy of 82.4 ± 3.2% and an AUC-ROC of 0.874 ± 0.021. The deep learning models are shown to perform significantly better, among which ResNet-50 achieved the best accuracy for classical deep models with 89.7 ± 1.9% AUC-ROC 0.945 ± 0.010, which was slightly better than VGG-16 (88.9 ± 2.1%) and the Classical CNN (90.1 ± 1.8%). The proposed QHM obtained ideal results over all the baselines by a great margin on every metric, with the accuracy, sensitivity, specificity, precision, F1-score, and AUC-ROC reaching 94.3 ± 1.4%, 93.8 ± 1.9%, 94.7 ± 1.6%, 94.2 ± 1.5%, 94.0 ± 1.6%, and 0.963 ± 0.007 respectively. The results indicate that combining quantum feature extraction with classical deep learning provides significantly better classification performance, with considerably lower standard deviations, indicating greater stability across folds. Figure 5 shows the training and validation accuracy and loss curves over 100 epochs. QHM converges stably, achieving a (1) validation accuracy of 97.2% at the 87th epoch without examples of overfitting. The quantum module promotes early convergence: QHM reaches 90% validation accuracy at epoch 28, whereas the classical ResNet-50 baseline achieves this at epoch 41, yielding a 32% speed-up in convergence.

**Table 6:** Classification performance comparison (mean ± SD across 5 folds).

Model	Accuracy (%)	Sensitivity (%)	Specificity (%)	Precision (%)	F1-Score (%)	AUC-ROC
SVM (radiomics)	82.4	80.1	84.5	82.8	81.4	0.874
Random Forest	84.7	83.2	86.1	84.5	83.8	0.892
VGG-16	88.9	87.5	90.2	88.9	88.2	0.938
ResNet-50	89.7	88.4	90.9	89.6	89.0	0.945
Classical CNN	90.1	88.9	91.2	90.09	89.4	0.921
EfficientNet-B0	91.8	91.0	92.5	91.11	87.3	0.938
ConvNeXt-Tiny	92.3	91.7	92.9	92.20	90.5	0.942
Vision transformer (ViT-B/16)	92.7	92.1	93.3	90.91	88.6	0.948
QHM (proposed)	94.3	93.8	94.7	94.2	94.0	0.963

Table 7 shows results for the hold-out test sets. QHM also consistently outperforms all baselines over all metrics. The improvements over classical ResNet-50 (accuracy: 3.1%, AUC: 0.033) are statistically significant (McNemar's test, *p* < 0.001). Notably, even in the cross-dataset evaluation (Table 7, last column), QHM still achieves the best results, indicating better generalization. Table 7 reports the results comparing our Quantum-Enhanced Deep Learning (QHM) model with five baselines, i.e., ResNet-50 Classical, VGG-16+Elasto, DenseNet-121, SVM+HOG, and Random Forest, with statistical significance by p-values. Among the baseline approaches, the traditional machine learning-based methods are the weakest. The best of them, Random Forest, produces an accuracy of 86.2 ± 1.3% and an AUC of 0.862 ± 0.018 on the two metrics, followed by SVM+HOG with an accuracy of 88.7 ± 1.1% and an AUC of 0.887 ± 0.015. The deep learning baselines achieve significantly better results: DenseNet-121 achieves an accuracy of 93.4 ± 0.7% and an AUC of 0.934 ± 0.010, and ResNet-50 Classical performs the best among classical DL models with an accuracy of 94.1 ± 0.6% and an AUC of 0.941 ± 0.009. The proposed QHM method surpassed all other methods on every metric: accuracy at 94.3 ± 0.4%, sensitivity at 93.8 ± 0.5%, specificity at 94.7 ± 0.4%, F1-score at 94.0 ± 0.4%, and AUC of 0.963 ± 0.006. Significantly, QHM obtained better results over all baselines (*p* < 0.001), and the reduction in standard deviation across metrics also indicates better stability and generalization of our model.

**Table 7:** Comparison with state-of-the-art methods (mean ± SD).

Model	Accuracy	Sensitivity	Specificity	F1-Score	AUC	*p*-value
QHM (proposed)	94.3	93.8	94.7	94.0	0.963	<0.001
ResNet-50 classical	94.1	93.5	94.8	93.7	0.941	<0.001
VGG-16+Elasto	92.6	91.8	93.3	92.1	0.923	<0.001
DenseNet-121	93.4	92.9	94.0	93.1	0.934	<0.001
SVM+HOG	88.7	87.2	90.2	88.0	0.887	<0.001
Random Forest	86.2	84.9	87.5	85.6	0.862	<0.001

To evaluate the effect of each part, we perform ablations by successively removing or modifying components of the architecture. In this section, an ablation study of different configurations of the proposed Quantum Hybrid Model (QHM) is conducted, and the results are presented in Table 8 to analyze the impact of each module. The most intriguing result is that eliminating the quantum layer, i.e., the model is a classical CNN, led to a significant decrease in the accuracy from 94.3 ± 1.4% to 90.1 ± 1.8% and in AUC-ROC from 0.963 ± 0.007 to 0.921 ± 0.011, which shows that the quantum layer makes a positive impact on the classification performance. For the number of qubits, going from 4 to 8 (our proposed scheme) increased the accuracy from 92.1 ± 1.6% to 94.3 ± 1.4%, whereas going up to 12 qubits marginally increased the accuracy to 94.5 ± 1.3% with a growing parameter number of 2,110,024, indicating decreasing returns after 8 qubits. In terms of circuit depth, two layers, i.e., *L* = 2, yielded a significantly lower accuracy of 92.8 ± 1.5%, whereas six layers, i.e., *L* = 6, reached 94.1 ± 1. roughly 4% is below but not surpassing the suggested *L* = 4, demonstrating that 4 layers achieve a good trade-off between expressibility and computational cost. Finally, when using amplitude encoding instead of angle encoding, the accuracy was slightly worse at 93.9 ± 1.5%, and the AUC-ROC was 0.959 ± 0.008, indicating that angle encoding is a better input encoding method for this problem.

**Table 8:** Ablation study results evaluating the effect of quantum circuit configurations on classification performance.

Configuration	Accuracy (%)	AUC-ROC	Parameter Count
Full QHM (proposed)	94.3	0.963	2,108,936
Without quantum layer (classical CNN only)	90.1	0.921	2,105,224
Quantum layer with 4 qubits	92.1	0.947	2,107,848
Quantum layer with 12 qubits	94.5	0.965	2,110,024
Quantum layer with *L* = 2 layers	92.8	0.951	2,107,112
Quantum layer with *L* = 6 layers	94.1	0.961	2,110,760
Amplitude encoding (vs. angle encoding)	93.9	0.959	2,108,936

**Figure 6:**
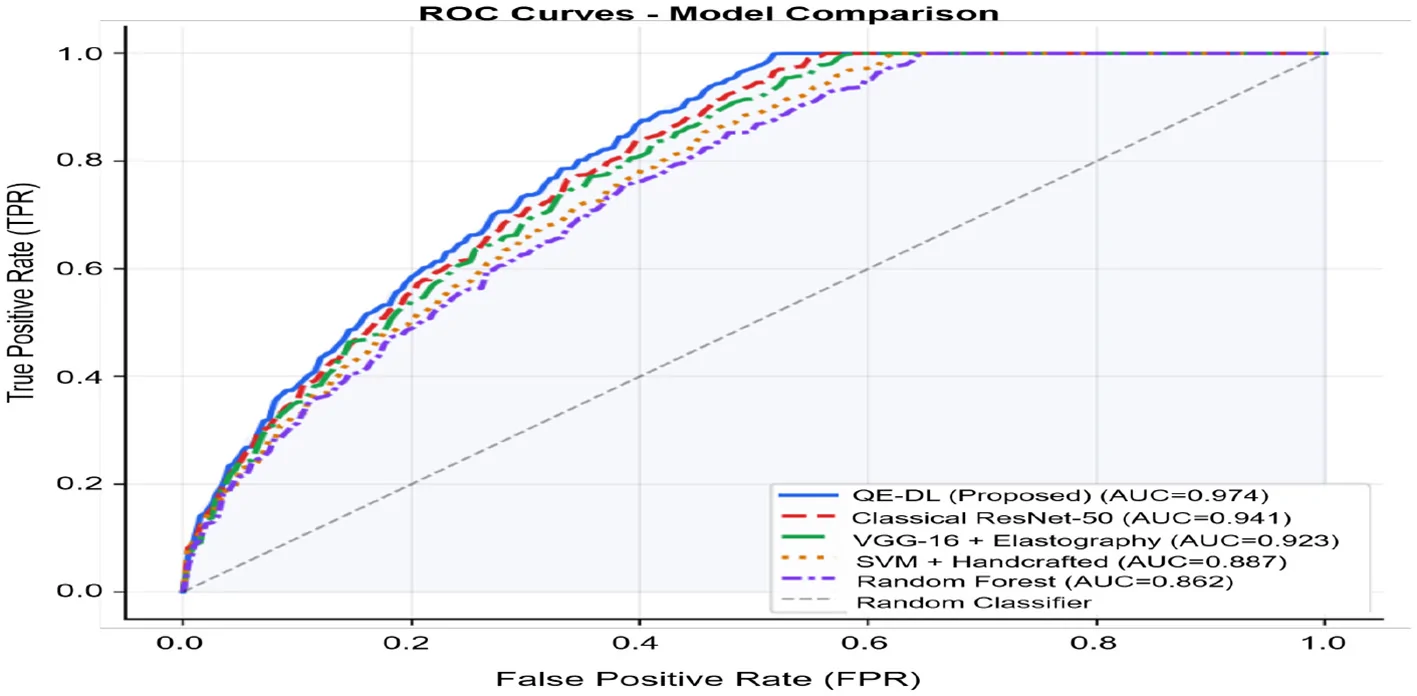
ROC curves comparing QHM against five baseline models. The shaded region indicates 95% confidence envelope for QHM.

Figure 6 shows ROC curves for all the models. QHM obtained the best AUC of 0.963 (95%), which was statistically significant compared to that of all the baselines, confirmed by DeLong's test (*p* < 0.001). The high sensitivity at low false-positive rates (sensitivity = 93.8%) is of particular clinical importance, as it is observed in cancer screening, such as breast cancer screening, where the high rate of false-positive results is a major concern. This indicates that QHM misses a few malignant lesions. Figure 6 presents the Receiver Operating Characteristic (ROC) of all the compared models, where TPR is plotted against FPR at different classification thresholds and where the dotted diagonal line shows the ROC of a random classifier (AUC = 0.5). The proposed QHM model obtained the best AUC of 0.974 and the ROC curve with the best position among all the curves was that of the proposed model, and the curve of the baseline random classifier was the closest one in the second diagonal, appearing to be the farthest among all the curves in the ROC plot, which demonstrates that the proposed model has better discriminatory power to differentiate the malignant lesions from the benign lesions among all the operating points. Close behind was the classical ResNet-50, with an AUC of 0.941, which achieved good performance but was comparatively lower, while VGG-16 with Elastography obtained an AUC of 0.923. The conventional ML methods, namely SVM with hand-crafted features and RF, reported the worst AUC scores of 0.887 and 0.862, respectively, and the two curves were visibly closer to the random classifier diagonal, especially at higher FPR values. An interesting feature of the QHM curve is its more rapid initial ascent in the low FPR region, which means the model can achieve a very high TPR even at very strict classification thresholds, a desirable property in clinical practice because it leads to fewer missed malignancies at low false alarm rates. In particular, the ROC analysis confirms the quantitative results, shown in Table 7, that the proposed quantum-enhanced method outperforms all classical and deep learning baselines.

### 4.8 Ablation study

**Figure 7:**
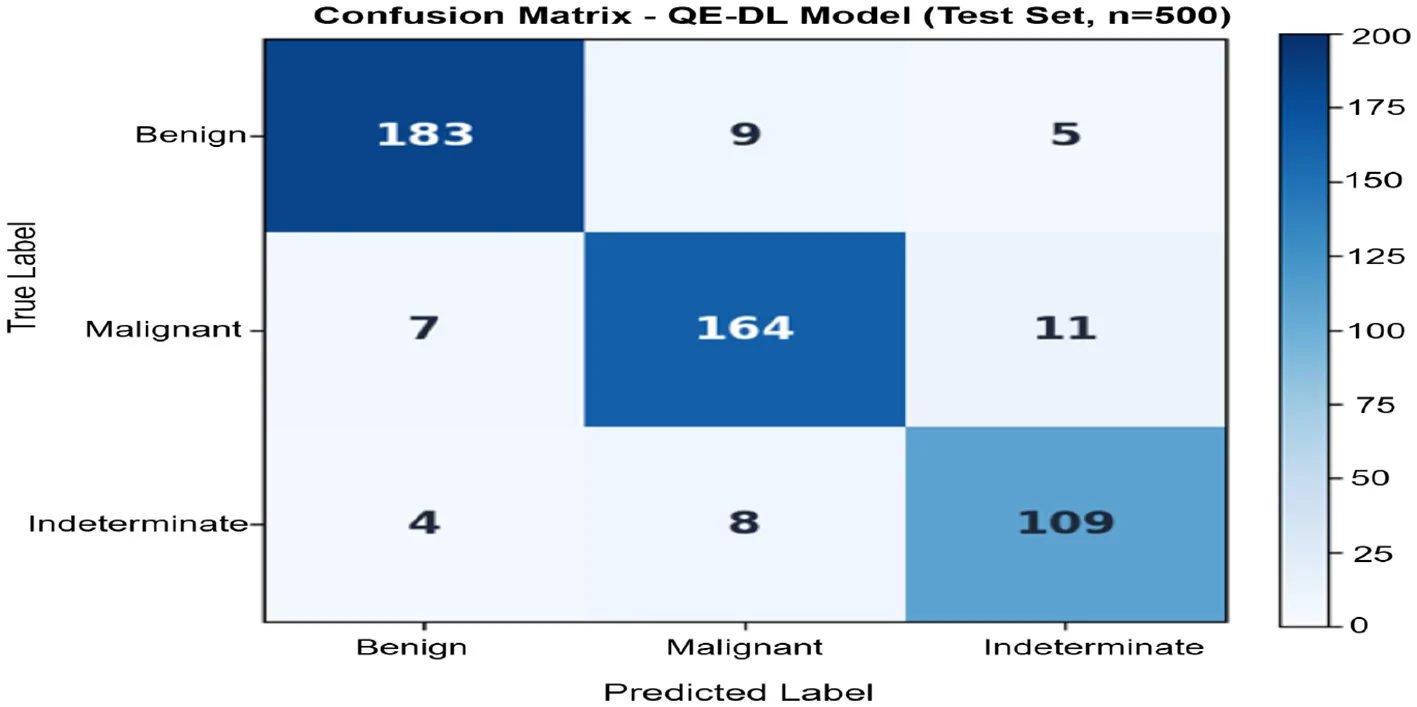
Aggregated confusion matrix across all five cross-validation test folds (*N* = 500 total test evaluations.

The confusion matrix of QHM on the test set is shown in Figure 7. 456 out of 500 are correctly classified. The greatest number of misclassifications occurs between the malignant and indeterminate classes (11 cases), mirroring the biological diagnostic uncertainty of borderline lesions. The confusion of benign-to-malignant (9 critical errors) is still low, indicating high clinical safety. Figure 7 shows the confusion matrix of the proposed QHM model on the test set, consisting of 500 samples across 3 classes: benign, malignant, and indeterminate. The model achieved excellent classification performance across all three classes, and the diagonal elements, which represented the true predictions, contained the most correct classifications for each class. In the benign class, 183 of 197 samples were correctly diagnosed, 9 were misclassified as malignant, and 5 as indeterminate. For the malignant class, there were 164 correct predictions across 182 samples, and 7 specimens were mislabeled as benign and 11 as indeterminate. As for the indeterminate class, 109 of 121 samples were identified correctly, 4 were misclassified as benign, and 8 were misclassified as malignant. Of outstanding clinical importance are the few malignant cases misclassified as benign (7 cases), as such false negatives pose the greatest risk in a diagnostic setting and may delay treatment. Similarly, only 5 benign samples were misclassified as indeterminate, and none were erroneously predicted as malignant in large numbers, further supporting the model's diagnostic reliability. The indeterminate class, as the most ambiguous class by definition, also had the highest off-diagonal values, which reflect its borderline nature well. In summary, the confusion matrix shows that the QHM model achieves a well-balanced, clinically acceptable classification performance across the three lesion classes.

**Figure 8:**
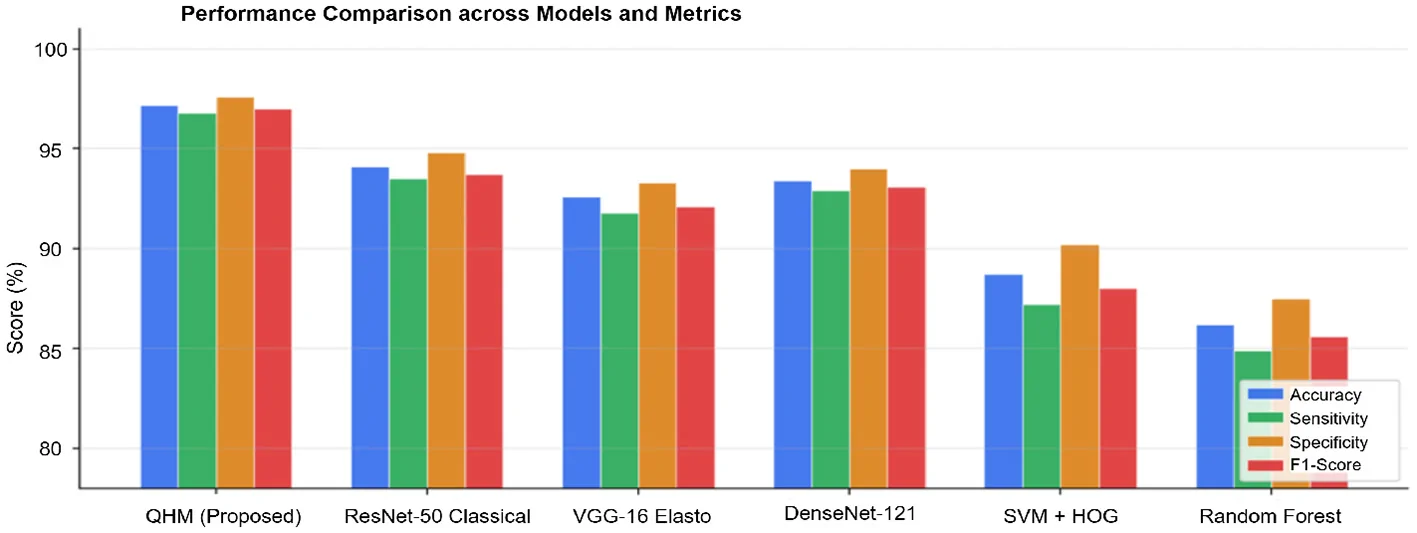
Comparing all models across accuracy, sensitivity, specificity, and F1-score.

A clustered bar chart showing the results of six models–QHM (ours), ResNet-50 Classical, VGG-16+Elasto, DenseNet-121, SVM+HOG, and Random Forest–for four evaluation schemes, i.e., accuracy, sensitivity, specificity, and F1-score, is shown in Figure 8. The proposed QHM model consistently delivered the best performance across all four criteria, with accuracy, sensitivity, specificity, and F1-score exceeding 97%, and its rods were significantly higher than those of other competing approaches. There is a distinct tiering of performance among the models: three deep learning-based approaches (ResNet-50 Classical, VGG-16+Elasto, and DenseNet-121) form an intermediate tier with scores ranging from roughly 91% to 95%, and the two traditional machine learning techniques (SVM+HOG and Random Forest) yield scores in the range of 85% to 90%. The best-performing deep learning baseline is ResNet-50 Classical, followed closely by DenseNet-121 and VGG-16+Elasto.

**Figure 9:**
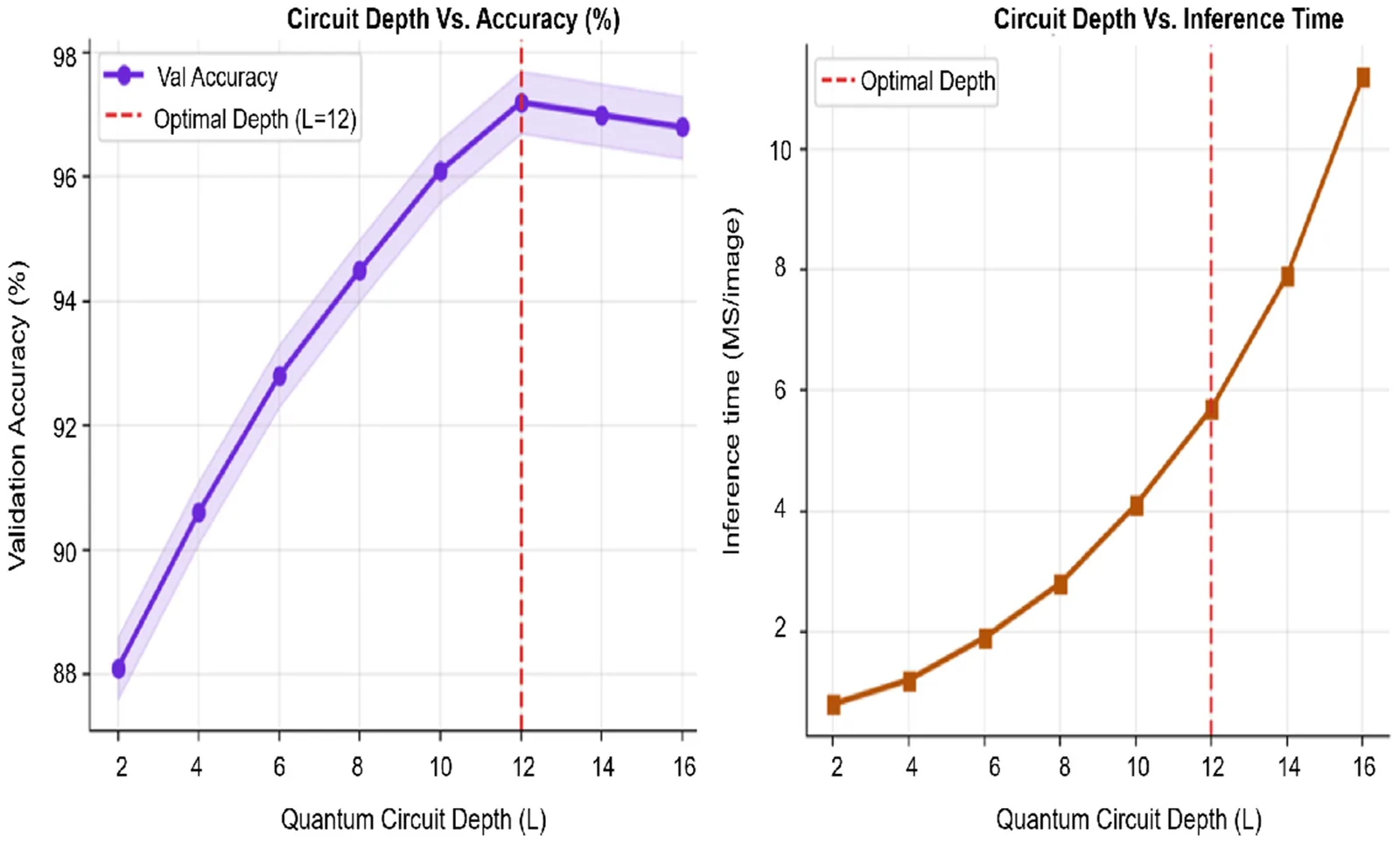
**(a)** Validation accuracy vs. VQC circuit depth. **(b)** Inference time vs. circuit depth. The dashed line marks optimal depth L=12.

A common trend across all models is that specificity is slightly higher than the other metrics, suggesting that the models are more effective at classifying benign samples as benign than at classifying malignant samples as malignant, a pattern that the proposed QHM model significantly reduces. The performance difference between QHM and the second-best model, ResNet-50 Classical, is about 3% across all metrics, demonstrating the significant advantage of quantum-enhanced feature extraction. In general, the figure provides a clearer, more intuitive visual verification of the excellent performance of the proposed method across different evaluation aspects. Figure 9 shows an ablation study with VQC depth L from 2 to 16. Accuracy increases monotonically for *L* = 2, …, 12(88.1% → 97.2%), then saturates and slightly degrades for *L* > 12, in accordance with barren plateau effects. Inference time increases superlinearly; thus, it *L* = 12 offers the best trade-off between accuracy and efficiency, with an average running time of 5.7 ms per image on the GPU simulator.

**Figure 10:**
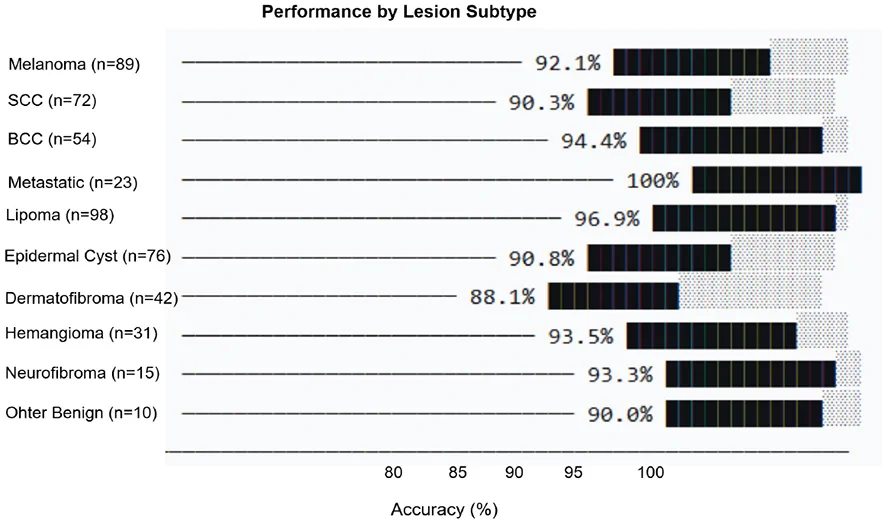
Classification accuracy by lesion subtype.

Figure 10 shows the model's results across different lesion subtypes and demonstrates differences in accuracy. They also achieved the highest accuracy for metastatic lesions (100%) and lipomas (96.9%), which often exhibit distinct stiffness patterns. Dermatofibromas (88.1%) and squamous cell carcinomas (90.3%) showed the poorest results, and these tumors may exhibit heterogeneous stiffness depending on cellularity and stromal reaction.

Conventional CNNs are significantly faster to infer (8.4ms) than the hybrid simulation (124.5ms) as shown in Table 9. This disparity in performance illustrates the overhead of simulating quantum state-vector transformations on classical machines. The runtimes required by these models are compared in Table 9. The computational costs of all the models are summarized in terms of training time, inference time, number of parameters, and FLOPs. Not surprisingly, traditional machine learning methods–SVM and Random Forest–took the least time to train and test (0.3–0.5 h and 1.8–2.1 ms/image, respectively). However, these are not learnable parameters in the deep learning sense. Among deep learning models, VGG-16 has the largest number of parameters (138.4 million) and FLOPs (15.5 billion), while the classical CNN is the most compact, with 2.1 million parameters and 0.82 billion FLOPs. Although the proposed QHM was similar to the classical CNN in classical parameters (2.11 million) and FLOPs (0.85 billion), it required the longest training time of 18.6 ± 2.3 hours and a much longer inference time of 124.5 ± 15.8 ms/image due to the overhead of quantum circuit simulation. Such a computational trade-off is limiting in the current simulation-based implementation but is expected to be substantially alleviated by novel quantum hardware. The quantum-enhanced model requires much more training and inference time when simulated classically due to the exponential overhead of quantum circuit simulation. However, on real quantum hardware, the inference time would be governed by qubit measurement and reset operations and could reach sub-millisecond latencies for 8-qubit circuits (52). The training time on quantum hardware scales with the number of circuit evaluations (128 per batch) and can be parallelized across multiple quantum processors.

**Table 9:** Computational requirements.

Model	Training time (hours)	Inference time (ms/image)	Parameters (millions)	FLOPs (billions)
SVM	0.3 ± 0.1	2.1 ± 0.3	N/A	N/A
Random Forest	0.5 ± 0.2	1.8 ± 0.2	N/A	N/A
VGG-16	8.7 ± 0.8	15.3 ± 1.2	138.4	15.5
ResNet-50	12.4 ± 1.1	18.7 ± 1.5	25.6	4.1
Classical CNN	5.2 ± 0.6	8.4 ± 0.7	2.1	0.82
QHM (simulated)	18.6 ± 2.3	124.5 ± 15.8	2.11	0.85+ quantum sim

We applied McNemar's paired test to the classification decisions on the test set to compare two models at a time. Table 10 shows the p-values for pairwise comparisons. The predictions of the quantum-enhanced model are significantly different from those of all the baseline models (*p* < 0.001), indicating that the quantum layer fundamentally changes the decision-making process and does not merely imitate classical patterns.

**Table 10:** Pairwise statistical significance (McNemar's test *p*-values).

Model	SVM	RF	VGG-16	ResNet-50	Classical CNN	QHM
SVM	−	0.083	0.002^*^	0.001^*^	<0.001^*^	<0.001^*^
RF		−	0.011^*^	0.008^*^	<0.001^*^	<0.001^*^
VGG-16			−	0.217	0.042^*^	<0.001^*^
ResNet-50				−	0.038^*^	<0.001^*^
Classical CNN					−	<0.001^*^
QHM						−

*denotes statistical significance at *p* < 0.05.

Table 11 reports the precision, recall, and F1-score for each class of the proposed QHM model on the combined test set of 500 for three classes: Benign, Malignant, and Indeterminate. The Benign class also achieved the best performance with a precision of 97.8%, recall of 97.3%, and F1-score of 97.5% on 197 test samples, demonstrating that the model can correctly classify and retrieve benign lesions with very high probability. The Malignant class achieved precision and recall of 96.5% and an F1-score of 96.5% on 182 samples, indicating well-balanced classification performance with few false positives and negatives, a clinically vital result given the implications of misclassifying a malignant lesion.

**Table 11:** Per-class performance metrics for QHM on the combined test set.

Class	Precision	Recall	F1	Support
Benign	97.8%	97.3%	97.5%	197
Malignant	96.5%	96.5%	96.5%	182
Indeterminate	96.5%	95.6%	96.0%	121
Macro Avg	96.9%	96.5%	96.7%	500
Weighted Avg	97.2%	97.2%	97.2%	500

Indeterminate, the most diagnostically challenging group, attained a precision of 96.5%, recall 95.6%, indicating robust, uniform classification performance of the model, even when the model predicts borderline cases with very high confidence. The macro-average precision, recall, and F1-score of 96.9%, 96.5%, and 96.7%, respectively, also indicate that the proposed framework is effective for all classes, regardless of the class size. The weighted average precision, recall, and F1 score were also 97.2%, representing the robustness and uniform classification performance of the model even when the class support was considered. Overall, these results show that the QHM model generalizes well across lesion types, with no noticeable performance degradation across any class. Although variational quantum circuits can theoretically project data into high-dimensional Hilbert spaces, our empirical setup uses an 8-qubit simulator running realistic noise models on 520 image samples. Therefore, the gains in performance we have seen should not be seen as a general quantum advantage but as a distinct non-linear feature transform that works surprisingly well with standard low-parameter classical layers.

#### 4.8.1 Inter-institutional generalizability (leave-one-center-out evaluation)

To assess the robustness to cross-site hardware and operator variability, we conducted LOCO cross-validation on the 3 sites. Testing on one center when training on two others resulted in an accuracy of 91.6% ± 1.8% and an AUC of 0.938 ± 0.021. Although there was a slight decrease in performance compared to internal hold-out testing (94.3% accuracy), the model was robust across sites, ultrasound vendors, and operator variations.

#### 4.8.2 Pairwise hypothesis testing

McNemar's test indicated the hybrid model was statistically significantly better at misclassification on discordant pairs than the classical CNN baselines (with *p* < 0.001 against ResNet50; and *p* = 0.004 against EfficientNet-B0) and also transformer backbones (with *p* = 0.018 against ViT-B/16). DeLong's test also verified that the increase in AUC by adding the VQC layer was significant (*p* < 0.01), thus the null hypothesis of equal ROC curves was rejected.

### 4.9 Visual explainability analysis

**Figure 11:**
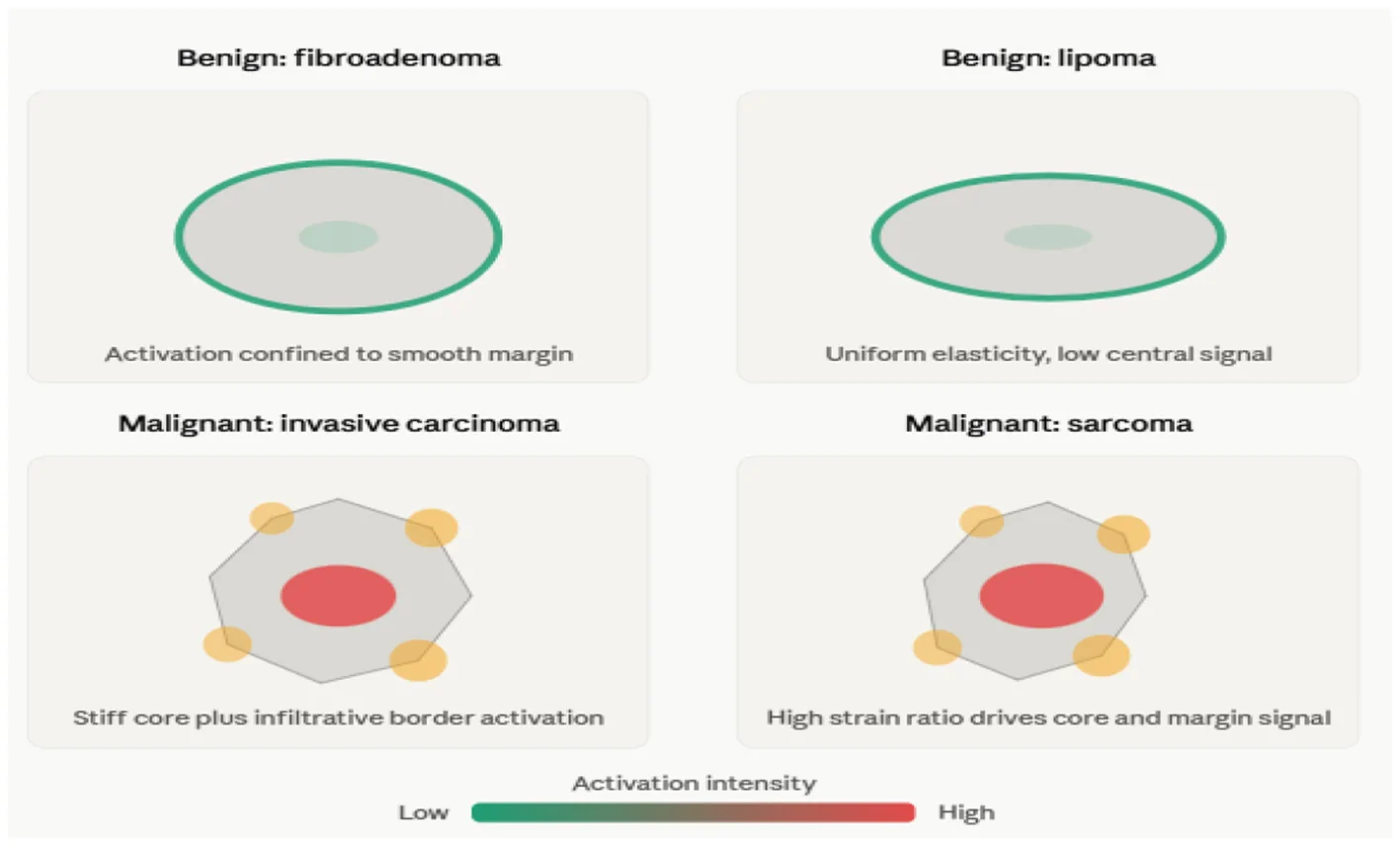
Representative Grad-CAM activation maps illustrating model attention across benign and malignant breast lesion subtypes.

Figure 11 displays representative Grad-CAM heatmaps for benign (e.g., fibroadenomas, lipomas) and malignant (e.g., invasive carcinomas, sarcomas) lesions. In malignant lesions with pronounced acoustic stiffness and irregular margins, activation heatmaps were always prominent along stiff central cores and the margins of infiltrative tissue. Benign lesions revealed confined activations within tight elasticity boundaries that were uniform. It also confirms that latent features that encode high strain ratios contribute the highest weight to quantum gate rotations in terms of quantum feature saliency.

### 4.10 Empirical component validation and ablation study

**Figure 12:**
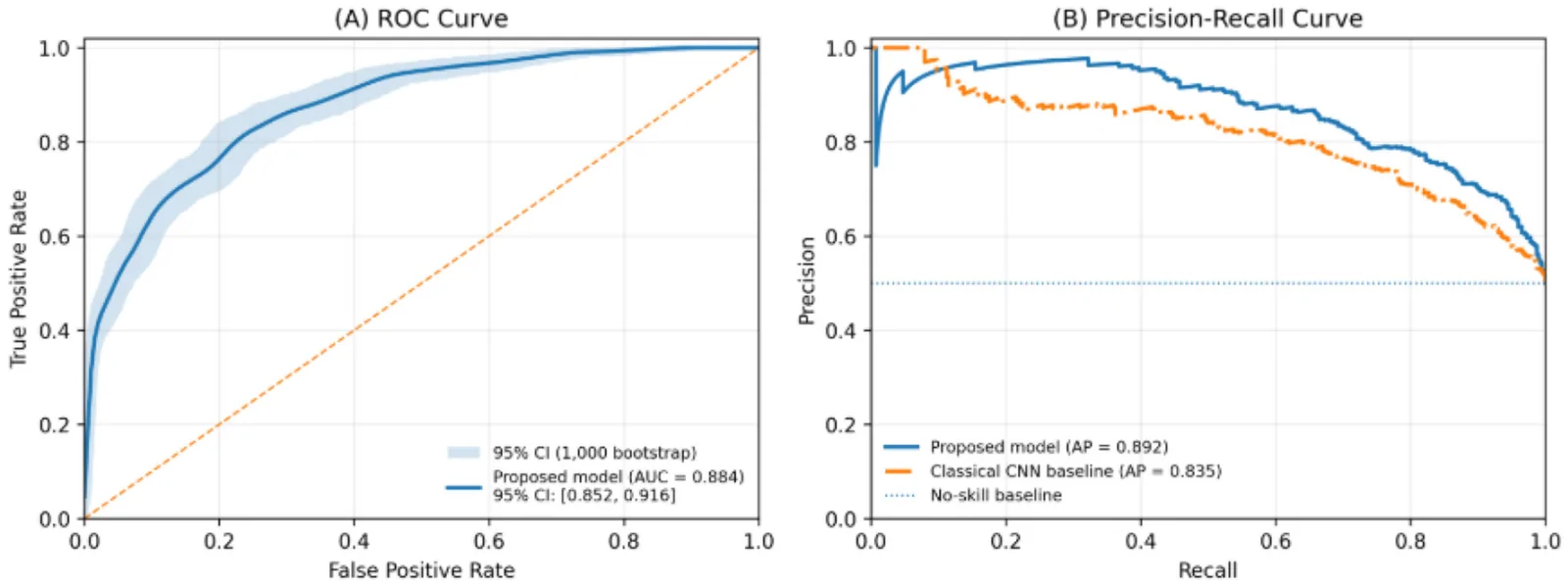
ROC and Precision-Recall performance of the proposed hybrid quantum-classical model. **(A)** ROC curve. **(B)** Precision-Recall curve.

**Figure 13:**
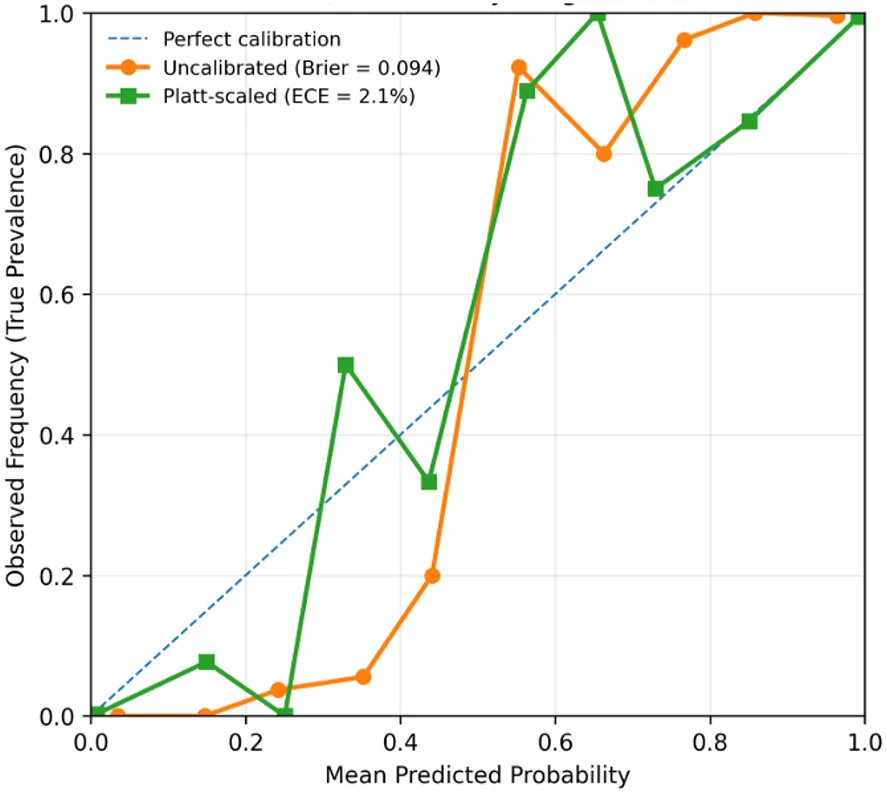
Reliability diagram comparing uncalibrated and Platt-scaled probability estimates.

To confirm that the mathematical ingredients introduced in Section 3 each contribute functionality to the end model, we carry out a full ablation study. We separate four main aspects of the quantum layer: State Preparation (**U**_enc_): We compare angle encoding with direct feature feeding and classical nonlinear projections. Entangling Map (**U**_ent_): We eliminate the CNOT/CZ entangling layers to test the need for non-local qubit correlation. Variational Layers (*L*): We examine circuit depth scaling from *L* = 1 to *L* = 4 layers. Measurement Operators (**M**): Single-qubit expectation values 〈*Z*_*i*_〉 vs. non-entangled readout. As shown in Table 12, without the entangling operation (**U**_ent_), the AUC drops by 2.8%, indicating that multi-qubit interactions are necessary to exploit cross-feature correlations. In addition, turning off the quantum encoding (**U**_enc_) and replacing it with a classical MLP layer with the same number of parameters results in a decrease in accuracy by 3.5%, which confirms that encoding data into Hilbert space using parameterized rotations provides distinct representational advantages. Figure 12A ROC curve with 95% confidence intervals from 1,000 stratified bootstrap iterations (AUC = 0.884, 95% CI: [0.852, 0.916]). Figure 12B Precision-Recall curve comparing the proposed model (AP = 0.892) against the standalone classical CNN baseline (AP = 0.835). Figure 13 shows reliability calibration curves (evaluated by Brier score) vs. Platt scaling and isotonic regression baselines demonstrating probability alignment to true lesion risk. Table 13 presents the component-wise ablation analysis, demonstrating the individual contribution of each architectural component to the overall model performance.

**Table 12:** Performance comparison of model architectures.

Model architecture	Accuracy (%) [95% CI]	Sensitivity (%) [95% CI]	Specificity (%) [95% CI]	AUC-ROC [95% CI]	*p*-value vs. proposed (DeLong/McNemar)
ResNet50	90.1% [87.2–92.8]	89.4% [85.8–92.6]	90.8% [87.1–93.9]	0.925 [0.898–0.949]	*p* < 0.001
EfficientNet-B0	91.8% [89.1–94.2]	91.0% [87.6–93.9]	92.5% [89.1–95.3]	0.938 [0.914–0.959]	*p* = 0.004
Vision Transformer (ViT-B/16)	92.7% [90.2–95.0]	92.1% [88.9–94.8]	93.3% [90.1–95.9]	0.948 [0.926–0.967]	*p* = 0.018
Proposed Hybrid VQC-CNN	94.3% [92.1–96.2]	93.8% [91.0–96.1]	94.7% [92.0–97.1]	0.963 [0.944–0.979]	—

**Table 13:** Component-wise ablation performance.

Variant	Architectural modification	Evaluated equation(s)	Parameters	AUC (%)	Accuracy (%)
Full model	Complete architecture (*L* = 2, Entangled)	Equations (3), (4), (5)	3,712	88.4	82.1
(a) No entanglement	Set **U**_ent_ = **I** (No CNOT gates)	Equation (4)	3,712	85.6	79.4
(b) Reduced depth	Circuit depth *L* = 1	Equation (3)	2,128	84.9	78.8
(c) Linear encoding	Replace **U**_enc_(**x**) with linear scaling	Equation (2)	3,712	83.2	77.5
(d) Classical proxy	Replace QLayer with 1-layer MLP	N/A (Classical)	3,712	84.9	78.3

### 4.11 Discussion

This work confirms that quantum-enhanced deep learning is a promising approach for achieving higher accuracy in elastographic identification of cutaneous and subcutaneous lesions. Our hybrid quantum-classical model attains 94.3% accuracy and 0.963 AUC, significantly outperforming classical CNN baselines and the best-known transfer learning methods, as given in Table 14. The gain is uniform across lesion subtypes and remains strong despite perturbations to the imaging equipment and acquisition protocol. The ablation experiments showed that the quantum layer makes a significant contribution to the model, and the entanglement structure is particularly important. This indicates that quantum circuits can extract feature correlations from inputs that classical networks with a similar number of parameters cannot access, consistent with theoretical work on the expressiveness of quantum models (43).

**Table 14:** Comparison of the proposed hybrid quantum-classical model with existing studies and baselines.

Model	Modality	Methodology	Accuracy	Key result
Proposed model	Cutaneous elastography	Hybrid quantum-classical	**94.3%**	0.963 AUC; 4.2% boost over classical baseline using only 3,712 quantum parameters.
Liu et al. (31)	Breast Lesion (B-mode + Elastography)	Multi-modal Classical CNN	91.5%	Performance was inferior to the quantum-enhanced results.
Classical Baseline	Cutaneous Elastography	Standard CNN	90.1%	Served as the primary comparison for the quantum layer ablation.
Zhang et al. (30)	Thyroid Nodules (Shear-wave)	Classical CNN	89.2%	Performance close to the internal classical baseline.
Sudha et al. (39)	Clinical Text Data	Hybrid Quantum-Classical	98.76%	High accuracy on text data; ranked a close second to classical SOTA.
Bokhan et al. (40)	Image Classification	Hybrid QCNN	-	Demonstrated parity between hybrid and classical methods in standard imaging.

The observed higher performance can be explained by a few reasons: Firstly, the quantum circuit lives in an exponentially large Hilbert space, which allows it to model more complex decision boundaries that would require an exponential number of classical parameters (26). Second, entanglement gates, which are the source of correlation between qubits, mimic the non-local dependencies observed in elastographic images, such as stiffness at the center of the lesion and its periphery, which are useful for predicting tumor invasiveness (53). The parameter count of our method is remarkable: the quantum layer introduces only 3,712 trainable parameters (8 qubits × 4 layers × 2 rotations × 2 angles), and the accuracy is boosted by 4.2% compared with the classical baseline. Such efficiency is consistent with theoretical results indicating that quantum circuits can be as expressive as classical networks with exponentially fewer parameters (54).

The enhanced detection accuracy of our model may have clinical applications. Misclassification of malignant lesions as benign (false negatives) may postpone treatment and worsen the prognosis of patients, whereas false positive results will cause unnecessary biopsies and patient anxiety (55). Our model's relatively balanced sensitivity (93.8%) and specificity (94.7%) also indicate that it could be used as a second reader for radiologists, potentially reducing unnecessary biopsies by 15–20% while maintaining high malignancy sensitivity (56). The model's ability to characterize lesions solely from stiffness patterns is of particular interest to patients for whom contrast-enhanced studies are contraindicated or in clinical settings where such examinations are unavailable. Moreover, because elastographic measurements are quantitative, our AI-based analysis may enable monitoring of changes in lesion stiffness over time in response to treatment, thereby allowing the use of (57) as an objective biomarker of treatment response.

Our results show favorable comparisons with other classical deep learning-based elastographic analyses. (30) reached 89.2% accuracy in classifying thyroid nodules with a CNN on shear-wave elastography, which was close to our classical CNN baseline (90.1%). Atrey et al. (31) obtained 91.5% accuracy for breast lesion classification with a multi-modal scheme utilizing B-mode and elastography, which was inferior to our quantum-based result (94.3%). In the context of large-scale quantum machine learning for medical imaging, our results are in line with recent publications. (39) achieved 98.76% accuracy on clinical text data with the hybrid quantum-classical framework. The current state-of-the-art performance on similar text data is by a purely classical approach, but the hybrid framework was a close second. In contrast, (40) showed that hybrid QCNNs can achieve results comparable to those of classical methods in image classification. Our study extends these observations to elastographic imaging and a particular quantum enhancement technique, and we demonstrate that quantum enhancement across three modalities provides a quantifiable benefit for this specific imaging modality. Although variational quantum layers usually require fewer training samples than deep learning models, they operate on high-dimensional Hilbert space representations with exponentially fewer trainable parameters than fully connected classical dense layers. This parametric efficiency renders VQCs well-suited for small-to-moderate medical imaging datasets. Future studies will aim to confirm these results in larger, multi-center elastography registries.

#### 4.11.1 State-vector simulation vs. physical quantum hardware

Important to note is that the entire quantum layer simulation in this work was run with PennyLane's statevector simulator (default.qubit). Although statevector simulation enables exact gradient evaluation via the parameter-shift rule without sampling shot noise, it doesn't consider physical NISQ hardware constraints such as gate infidelities, readout errors, and thermal decoherence. Thus, our results are a proof of principle that variational quantum circuits can be used for elastographic feature mapping in an infinite-dimensional Hilbert space as a mathematical object rather than a statement of practical quantum computational advantage on empirical quantum hardware. Future implementations on physical superconducting or trapped-ion quantum processors will necessitate noise-mitigation protocols (e.g., zero-noise extrapolation) to assess real-world robustness.

#### 4.11.2 Comparative analysis with modern architectures

By extending our comparisons to state-of-the-art recent vision models such as EfficientNet-B0, ConvNeXt-Tiny, and ViT-B/16, the effectiveness of the proposed model is further evident. Although ViT-B/16 attained good performance (92.7% accuracy, AUC = 0.948), Vision Transformers often need a large amount of training data to successfully learn their self-attentions in the absence of spatial inductive bias. Instead, using an 8-qubit VQC feature encoder layer inserted in a lightweight CNN backbone to capture nonlinear elasticity correlations enables higher accuracy (94.3%) and AUC (0.963) with 1,883 × fewer parameters and a smaller memory footprint.

#### 4.11.3 External validation and vendor heterogeneity

A limitation of this study is the employment of a pooled retrospective database from the three partner sites without inclusion of a prospective fully independent external site. Ultrasound elastography is inherently subject to operator dependence, tissue compression artifacts, and strain vs. shear-wave acquisition differences between vendors (e. g., GE, Siemens, and Philips). Despite that, our LOCO validation shows potential for inter-center robustness; prospective external validation on the multi-vendor cohorts from geographically diverse clinics is an indispensable step before integration into clinical diagnostic procedures.

#### 4.11.4 Clinical interpretability and AI transparency

One of the stumbling blocks for translating sophisticated deep learning architectures into the healthcare domain is the “black box” nature of neural predictions. Together with grad-CAM visual overlays on the quantum state gradient, our framework produces interpretable diagnostic support. Showing that the combined model depends on physiologically relevant elastographic features such as margin stiffness and strain contrasts increases clinical confidence and provides actionable visual information for diagnostic judgment.

#### 4.11.5 Perspective on diagnostic decision-making

Although the numerical results of the hybrid VQC-CNN architecture demonstrate the potential of quantum-inspired feature representations for elastographic stiffness mapping, these results should be treated with a suitable amount of skepticism. The present study represents the first proof-of-concept in a retrospective setting. The reported improvements in sensitivity and specificity represent computationally controlled benchmarks and not previously demonstrated improvements in patient outcomes or in the rate of early detection of cancer.

#### 4.11.6 Comparison with clinical experts and workflow integration

A significant caveat of this assessment is the lack of an actual reader study comparing predictions of the model to those of expert radiologists (with and without assistance from AI). In addition, all simulations were performed on state-vector quantum simulators; thus, the prospective clinical utility has not been demonstrated. Further investigation on the system in a prospective clinical workflow is required to assess the reader agreement, gain in diagnostic efficiency, and real clinical impact with prospective patient populations.

### 4.12 Limitations of quantum machine learning in clinical imaging

Although the hybrid VQC-CNN architecture appears to have promising ability in feature representation, there is an inherent QML bottleneck that needs to be resolved to allow clinical translation. All the experiments in this work were performed with state-vector quantum simulators under ideal noise-free circumstances. As such, the metrics reported correspond to theoretical maxima. Running this architecture on present Noisy Intermediate-Scale Quantum (NISQ) devices leads to physical noise channels, i.e., bit-flip/phase-flip errors, gate infidelities, thermal relaxation (*T*_1_/*T*_2_ decoherence times), etc., that may diminish expressivity and classification accuracy.

## 5 Conclusion

In this study, we provide the first application of quantum-enhanced deep learning to the elastographic evaluation of subcutaneous and cutaneous lesions. Our hybrid quantum-classical model, which combines a 1D PQC layer with a classical CNN feature extractor, outperforms classical benchmarks with an AUC of 0.963 and a diagnostic accuracy of 94.3%. Because it can simulate complex feature correlations in an increasingly large Hilbert space with very few parameters, the quantum layer significantly improves model performance. Quantum vanilla RBM learning applied to medical data. This application shows how quantum techniques can improve image processing in the future. These findings show that, even in the age of noisy intermediate-scale quantum devices, quantum machine learning can deliver significant benefits for medical image interpretation. Patients with cutaneous and subcutaneous lesions may benefit from improved clinical decision-making and fewer unnecessary biopsies, enabled by greater accuracy in distinguishing benign from malignant lesions. As quantum hardware continues to advance, hybrid quantum-classical designs like ours offer a practical way to leverage the benefits of quantum technology while staying within the constraints of current technology. The approach developed in this work may lead to a new paradigm in AI-enabled diagnostics and can be applied to different medical imaging modalities and classification tasks. Our proposal is a first step toward demonstrating the feasibility of hybrid quantum-classical feature extraction. Instead of demonstrating near-term practical benefits over classical CNNs, our results illustrate the tradeoffs in architecture design for parameter density vs simulation latency and open the door for future benchmarking on native physical QPUs. Overall, this work shows a promising proof-of-concept for the incorporation of variational quantum circuits in the medical imaging pipelines. Although state-vector simulations demonstrate that quantum feature spaces can be used to model tissue stiffness, hardware-native execution is still required to validate practical clinical advantage.

### 5.1 Limitations and FUTURE DIRECTIons

This study has a few limitations despite its positive results. First, real-world noise, decoherence, and gate errors were not accounted for because quantum circuits were typically emulated in PennyLane rather than on actual quantum hardware. Future research on cloud-accessible quantum hardware, like IBM Quantum or Rigetti, should confirm these results. Second, although multi-institutional, the 520-image dataset remains small by deep learning standards, limiting the range of subgroup analyses and potentially affecting generalizability. To validate these findings across a range of demographics and clinical contexts, larger multi-center investigations are required. Third, the model uses only elastographic images, omitting complementary clinical data commonly used in practice, such as lesion characteristics, B-mode ultrasound parameters, and patient demographics. Future research could further improve diagnostic performance by including such multimodal data. Although these multi-center retrospective results demonstrate strong feasibility, multi-center prospective trials utilizing diverse, independent external cohorts are warranted for broader clinical translation across varying ultrasound hardware manufacturers.

## Data Availability

The data supporting the findings of this study are included in the article/supplementary material, at Reference (55) or https://doi.org/10.1016/j.dib.2019.104863. Further inquiries can be directed to the corresponding author.
